# Regulation of Cdc42 signaling by the dopamine D2 receptor in a mouse model of Parkinson’s disease

**DOI:** 10.1111/acel.13588

**Published:** 2022-04-12

**Authors:** Li Ying, Jinlan Zhao, Yingshan Ye, Yutong Liu, Bin Xiao, Tao Xue, Hangfei Zhu, Yue Wu, Jing He, Sifei Qin, Yong Jiang, Fukun Guo, Lin Zhang, Nuyun Liu, Lu Zhang

**Affiliations:** ^1^ Key Laboratory of Functional Proteomics of Guangdong Province Key Laboratory of Mental Health of the Ministry of Education School of Basic Medical Sciences Pediatric Center of Zhujiang Hospital Center for Orthopaedic Surgery of the Third Affiliated Hospital Southern Medical University Guangzhou China; ^2^ Division of Experimental Hematology and Cancer Biology Children's Hospital Research Foundation Cincinnati Ohio USA; ^3^ Department of Histology and Embryology NMPA Key Laboratory for Safety Evaluation of Cosmetics Key Laboratory of Construction and Detection in Tissue Engineering of Guangdong Province School of Basic Medical Sciences Center for Orthopaedic Surgery of the Third Affiliated Hospital Southern Medical University Guangzhou China; ^4^ Laboratory Animal Center Elderly Health Services Research Center Southern Medical University Guangzhou China

**Keywords:** caudate putamen, Cdc42, dopamine D2 receptor, medium spiny neurons, Parkinson's disease

## Abstract

Substantial spine loss in striatal medium spiny neurons (MSNs) and abnormal behaviors are common features of Parkinson's disease (PD). The caudate putamen (CPu) mainly contains MSNs expressing dopamine D1 receptor (dMSNs) and dopamine D2 receptor (iMSNs) exerting critical effects on motor and cognition behavior. However, the molecular mechanisms contributing to spine loss and abnormal behaviors in dMSNs and iMSNs under parkinsonian state remain unknown. In the present study, we revealed that Cell division control protein 42 (Cdc42) signaling was significantly decreased in the caudate putamen (CPu) in parkinsonian mice. In addition, overexpression of constitutively active Cdc42 in the CPu reversed spine abnormalities and improved the behavior deficits in parkinsonian mice. Utilizing conditional dopamine D1 receptor (D1R) or D2 receptor (D2R) knockout mice, we found that such a decrease under parkinsonian state was further reduced by conditional knockout of the D2R but not D1R. Moreover, the thin spine loss in iMSNs and deficits in motor coordination and cognition induced by conditional knockout of D2R were reversed by overexpression of constitutively active Cdc42 in the CPu. Additionally, conditional knockout of Cdc42 from D2R‐positive neurons in the CPu was sufficient to induce spine and behavior deficits similar to those observed in parkinsonian mice. Overall, our results indicate that impaired Cdc42 signaling regulated by D2R plays an important role in spine loss and behavioral deficits in PD.

## Graphical abstract

Substantial spine loss in striatal medium spiny neurons and abnormal behaviors are common features of Parkinson's disease. Utilizing conditional dopamine D1 receptor or D2 receptor knockout mice, our results demonstrate that impaired Cdc42 signaling, regulated by dopamine D2 receptor but not D1 receptor, plays an important role in spine loss and behavioral deficits in a Parkinson's disease mouse model.

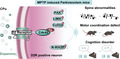

## 1 INTRODUCTION

Parkinson's disease (PD) is a neurodegenerative disease characterized by motor coordination deficits and cognitive impairment, which are caused by the premature death of dopaminergic neurons innervating the striatum (Ascherio & Schwarzschild, 2016). The striatum, an important substrate implicated in psychomotor functions, mainly receives dopaminergic afferents from the substantia nigra pars compacta (SNpc) and forms two parallel efferent pathways, including the striatonigral direct pathway and the striatopallidal indirect pathway (Darvas & Palmiter, 2010). Striatonigral neurons in the direct pathway primarily express the dopamine D1 receptor (D1R) and the neuropeptide dynorphin (DYN), and striatopallidal neurons in the indirect pathway express the D2 receptor (D2R) and enkephalin (ENK). Numerous lines of evidence indicate that the imbalance of the two pathways is responsible for the abnormal behavior in parkinsonian animals (Suarez et al., 2014). Additionally, different portions of the striatum have been neuroanatomically identified as serving different functions: the ventral portion, namely the nucleus accumbens (NAc), is involved in reward and reinforcement; the dorsal portion, designated the caudate putamen (CPu), is fundamental to cognition and motor control (Graybiel, 2000). Numerous studies have shown that dopaminergic deficiency in the CPu contributes to cognitive decline and motor deficits in PD (McCoy et al., 2019). For instance, intra‐CPu infusion of a D1R agonist or D2R agonist efficiently improved motor dysfunction in 6‐OHDA‐induced PD rats (M. Guo et al., 2021). Additionally, the enhancement of dopaminergic signals in the CPu was consistent with improvements in both motor function and cognition of PD participants (Darvas et al., 2014; McCoy et al., 2019). The most common cell type in the CPu is medium spiny neurons (MSNs), which bear the brunt of reduced dopaminergic innervation in PD (Alberquilla et al., 2020; Ascherio & Schwarzschild, 2016).

Dendritic spines are highly dynamic structures that are deeply associated with synaptic plasticity, learning, memory, and behavioral output (Du et al., 2020; Zolochevska & Taglialatela, 2020). MSNs are generally accepted to undergo a significant loss of dendritic spines in various PD models and PD patients (Beeler et al., 2012). Postmortem study has demonstrated dendrites and spine density on MSNs were dramatically decreased in PD patients (Zaja‐Milatovic et al., 2005). In parallel, subsequence studies revealed that MSNs spine density was reduced in animal models of PD (Beeler et al., 2012). Additionally, evidence from PD models has shown a significant loss of dendritic spines from both MSNs expressing dopamine D1R (direct MSNs, dMSNs) and MSNs expressing the dopamine D2R (indirect MSNs, iMSNs) within the striatum (Suarez et al., 2014). Furthermore, Surmeier and his colleagues recently demonstrated that the loss of dMSNs dendritic spines lagged behind that of iMSNs dendritic spines in parkinsonian mice (Graves & Surmeier, 2019). Both the D1R and D2R signaling pathways play essential roles in PD‐associated spine loss and behavioral changes (Suarez et al., 2016). For example, the degeneration of dopaminergic neurons induces an imbalance in D1R and D2R signaling in the striatum, thus impairing motor behavior. The imbalance is due to either aberrant neural excitability of dMSNs and iMSNs or aberrant striatal plasticity following dopamine depletion (Suarez et al., 2016). In addition, recent studies have demonstrated that blocking D1R signaling in the motor cortex increases spine loss, while blocking dopamine D2R signaling promotes spine formation in mouse models of PD and that this atypical spine remodeling contributes to the motor memory impairment associated with PD (Guo et al., 2015). Further studies have shown that reduced dopamine signaling induces motor and cognitive impairments (Morgan et al., 2015), and that deletion of D2Rs within the striatum results in dyskinesia similar to that observed in PD models (Bello et al., 2017). Moreover, the restoration of dopaminergic signaling in the dorsolateral striatum is able to reverse the cognitive impairments uncovered in dopamine‐deficient mice (Darvas & Palmiter, 2009). However, despite the myriad data showing the importance of D1R and D2R signaling in PD, knowledge regarding the precise molecular mechanisms of D1R and D2R signaling in the regulation of spine loss and behavioral output associated with PD is limited.

Cell division control protein 42 (Cdc42) is a member of Rho GTPases and plays an important role in modulating spine plasticity (Scott et al., 2003). Similar to other small GTPases, Cdc42 cycles between the GDP‐bound inactive state and the GTP‐bound active state (Haspel et al., 2021). Two states are primarily controlled by two opposing enzymes: (i) guanine nucleotide exchange factors (GEFs), which catalyze the exchange of bound GDP for GTP and shift Cdc42 into the activated state (active Cdc42), and (ii) GTPase‐activating proteins (GAPs), which accelerate GTP hydrolysis, rendering Cdc42 into the inactivated state (inactive Cdc42) (Haspel et al., 2021). Only active Cdc42 interacts with downstream effectors to elicit cellular processes (Haspel et al., 2021; Kim et al., 2014). Many studies indicate that Cdc42 underpins dendritic spine morphogenesis through the downstream neural Wiskott–Aldrich syndrome protein (N‐WASP) signaling pathway and p21‐activated kinase (PAK) signaling pathways (Scott et al., 2003). For example, inhibition of Cdc42‐PAK pathway impairs spine structural and functional plasticity in hippocampal cultured slices, and the downregulation of Cdc42‐N‐WASP pathway impairs spine formation (Wegner et al., 2008). Abnormal Cdc42 signaling has been linked to cognitive impairments and aberrant spine remodeling in certain psychiatric and neurodegenerative disorders, including schizophrenia and Alzheimer's disease (Eira et al., 2016). For example, decreased Cdc42 activity impairs remote memory recall (Kim et al., 2014) and contributes to the reduction in dendritic spines in layer 3 pyramidal neurons in schizophrenia (Ide & Lewis, 2010). Notably, several lines of evidence suggest the involvement of Cdc42 in PD. First, the expression of the *Cdc42* gene is consistently reduced in three brain regions (Brodmann's area, putamen, and substantia nigra) in postmortem brain tissues from patients with PD (Zhang et al., 2005). Second, genomic analyses of inherited PD have demonstrated that the downregulation of *Cdc42* mRNA is involved in neural apoptosis (Chi et al., 2018; Habib et al., 2018). Third, a protein array analysis indicates that Cdc42 effector proteins (Cdc42EP2) could bind to α‐synuclein, revealing a mechanism underlying the neuropathology of PD (Schnack et al., 2008). Thus, we hypothesized that Cdc42 may be crucially involved in Parkinson's disease‐like behavior. The goal of the present study was to investigate whether the reduction in Cdc42 signaling in the CPu is involved in PD‐like behavior and, if so, whether D1Rs or D2Rs in the CPu are involved in the regulation of Cdc42 signaling. We found that Cdc42 signaling was decreased in the CPu in parkinsonian mice, accompanied by significant spine loss and impairments in motor coordination and cognitive function. Moreover, overexpression of constitutively active Cdc42 reversed spine loss in the CPu and improved the deficits in motor coordination and cognition observed in parkinsonian mice, suggesting that Cdc42 signaling plays an important role in the spine abnormalities and behavioral deficits associated with PD. In addition, using CPu‐*Drd1*KO or CPu‐*Drd2*KO mice, we found that Cdc42 signaling was regulated by D2R rather than by D1R under parkinsonian state and that overexpression of constitutively active Cdc42 reversed the D2R knockout‐induced spine abnormalities and behavioral deficits. Moreover, using *Drd2*‐Cdc42KO mice, we found that conditional knockout of Cdc42 in striatal D2R‐positive neurons induced spine abnormalities as well as deficits in motor coordination and cognition, similar to those observed in parkinsonian mice. Taken together, our results provide the first evidence that Cdc42 signaling, regulated by D2R in the CPu, plays an important role in mediating spine abnormalities and motor and cognitive deficits in PD.

## 2 RESULTS

### 2.1 Involvement of Cdc42 activity in regulating spine plasticity in parkinsonian mice

**FIGURE 1:**
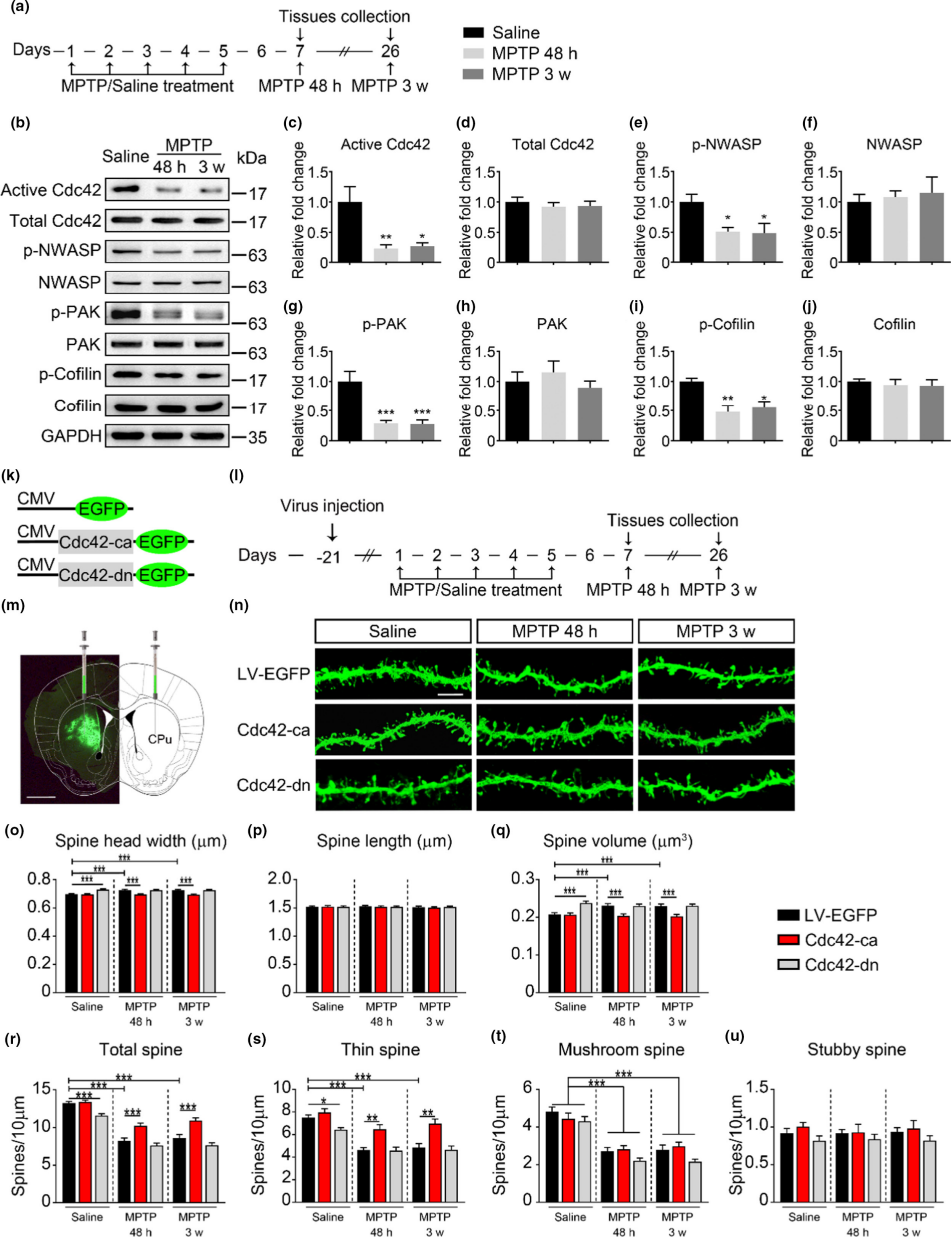
Involvement of Cdc42 activity in regulating spine plasticity in parkinsonian mice. (a) Timeline for successive intraperitoneal injection of MPTP or saline and tissues collection. (b‐j) Representative western blots (b) and statistical analyses (right) assessing the level of Cdc42 activity and its downstream effectors in the CPu 48 h and 3 weeks after the final injection of MPTP. The activity of Cdc42 (c) and the phosphorylated forms of N‐WASP (e), PAK (g), and Cofilin (i) in the CPu were decreased 48 h and 3 weeks after the final injection of MPTP. Total Cdc42 (d), N‐WASP (f), PAK (h), and Cofilin (j) were not altered by MPTP at the indicated time points (*n* = 6 mice/group). (k) Schematic diagram of the construction of lentiviruses containing EGFP alone (LV‐EGFP), constitutively active Cdc42 (Cdc42‐ca), or dominant negative Cdc42 (Cdc42‐dn) fused with EGFP driven by the CMV promoter. (l) Timeline for analysis of the spine structure from mice injected with Cdc42 mutant constructs. (m) Representative image of coronal sections from the mouse brain showing lentiviral EGFP expression within the CPu 3 weeks after stereotaxic injection. Scale bars, 1 mm. (n) Representative confocal images from dendritic spines transfected with Cdc42 mutants at both 48 h and 3 weeks. Scale bars, 5 μm. (o–q) Statistical analysis of average spine head width (o) spine length (p) and spine volume (q) in MSNs from virus‐transfected dendrites in the CPu at both 48 h and 3 weeks. (r–u) Statistical analysis of dendritic spine density, including total (r), thin (s), mushroom (t), and stubby (u) spine subtypes at both 48 h and 3 weeks. The data represent the mean ± *SEM*, and the normal saline group was set to 1 for the quantitative analysis, *p < 0.05, **p < 0.001, ***p < 0.0001, one‐way ANOVA (c–j), followed by Bonferroni correction for multiple comparisons, and two‐way ANOVA (o–u), followed by Bonferroni correction for multiple comparisons

To investigate whether Cdc42 signaling is involved in the pathophysiology of parkinsonian mice, Cdc42 activity in the CPu was measured both 48 h and 3 weeks after the final injection (Figure 1a). Active Cdc42 but not total Cdc42 was significantly decreased in the CPu of parkinsonian mice (Figure 1b,c,d). The downstream targets of Cdc42 signaling, including the phosphorylation levels of N‐WASP, PAK, and Cofilin, were all decreased (Figure 1b,e,g,i). Total Cdc42, N‐WASP, PAK, and Cofilin were unaltered by 1‐methyl‐4phenyl‐1,2,3,6‐tetrahydropyridine (MPTP) at the indicated time points (Figure 1b,d,f,h,j). These findings suggest that Cdc42 signaling in the CPu is significantly suppressed in parkinsonian mice.

To examine whether the reduction in Cdc42 activity in parkinsonian mice is responsible for spine alteration in the CPu, we constructed several lentiviruses containing Cdc42 mutants, including a constitutively active mutant Cdc42‐ca and a dominant negative mutant Cdc42‐dn (Figure 1k). Constitutively active Cdc42 mutants (Cdc42‐ca) are resistant to GTP hydrolysis and thus maintain Cdc42 in active state, while dominant negative mutants (Cdc42‐dn) sequester GEFs and inhibit Cdc42 activity (Haspel et al., 2021). Mice were bilaterally injected with lentiviruses into the CPu, and brain tissues were collected 48 h and 3 weeks after intraperitoneal injection (Figure 1l). Immunofluorescence staining confirmed that the virus was confined to the CPu and spread throughout approximately 50.05% of the CPu (Figure S2a). Three weeks after virus injection, the activities of Cdc42 and its downstream proteins (N‐WASP, PAK, and Cofilin) were assessed. The results showed that Cdc42‐ca efficiently increased Cdc42 and its downstream signaling activities, and Cdc42‐dn decreased Cdc42 and its downstream signaling activity (Figure S2). NeuronStudio was used to process the morphological features of dendritic spines. Similar to the stereology obtaining three‐dimensional parameters of the structure based on the measurements of two‐dimensional slices, NeuronStudio with the rayburst algorithm allows for 3D reconstruction of dendritic spines from multiple confocal stacks in a spatial range (Rodriguez et al., 2006). We found that MPTP treatment significantly increased spine head width (Figure 1n,o) and volume (Figure 1n,q) without changes in the average spine length (Figure 1n,p) both 48 h and 3 weeks after the final injection of MPTP. Noting that Cdc42 activity was decreased by MPTP treatment (Figure 1b,c), overexpression of Cdc42‐dn increased spine head width and spine volume, whereas overexpression of Cdc42‐ca significantly improved the enlarged spine heads and spine volume (Figure 1o,q). Further decreasing Cdc42 activity in MPTP‐treated mice by overexpression of Cdc42‐dn did not exacerbate MPTP‐induced increase in spine head width and volume (Figure 1o,q), suggesting that MPTP‐induced decrease in Cdc42 activity is sufficient to increased spine head width and volume. We further found that MPTP treatment reduced the total spine density (Figure 1n,r) and that this decrease was mainly due to the loss of thin (Figure 1n,s) and mushroom spines (Figure 1n,t) rather than stubby spines (Figure 1n,u). Overexpression of Cdc42‐dn reduced total spine and thin spine density, whereas overexpression of Cdc42‐ca partially reversed the MPTP‐induced reduction in total spine and thin spine density (Figure 1r–s). Overexpression of Cdc42‐dn did not further decrease total and thin spine density in MPTP‐treated mice (Figure 1r–s). These results indicate that the impaired Cdc42 activity is important for the changes in the spine head, spine volume, and thin spine density in parkinsonian mice.

### 2.2 Involvement of Cdc42 activity in regulating motor deficits and cognitive impairment in parkinsonian mice

**FIGURE 2:**
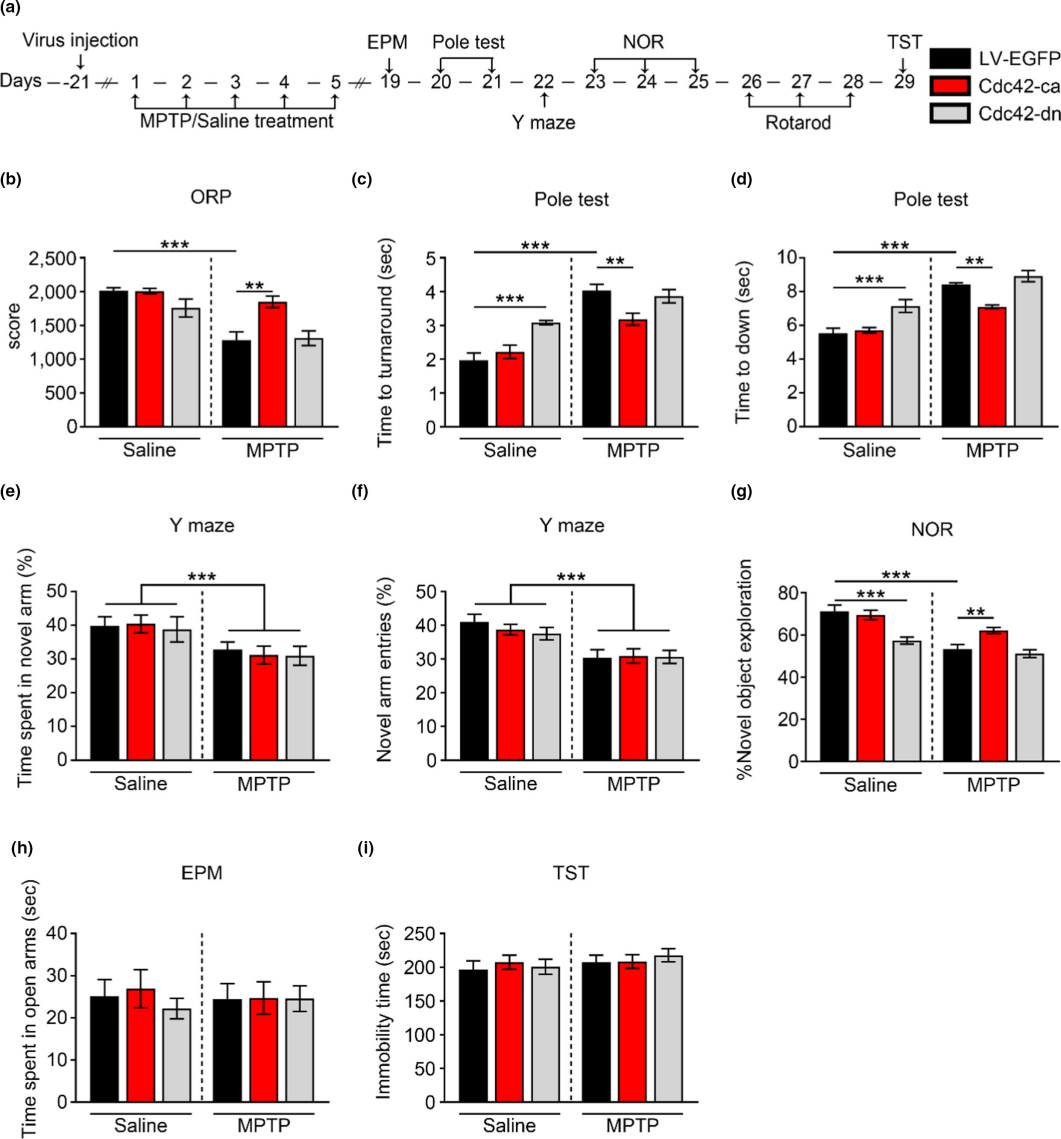
Involvement of Cdc42 activity in regulating motor deficits and cognitive impairment in parkinsonian mice. (a) Timeline for analysis of the behavior of the mice injected with the Cdc42 mutant followed by MPTP or saline treatment. (b–i) Quantification of behavioral parameters. Movement distance in the performance score in overall rotarod performance (ORP) (b), and latency to turn around (c) and land on the ground (d) in the pole test; (e–g) Cognitive function was measured by the percentage of time spent in the novel arm (e) and visits to the novel arm (f) in the Y‐maze and percentage of time exploring the novel object in the novel object recognition (NOR) task (g); (h and i) Anxiety and depression‐like behavior were quantified by an Elevated plus‐maze (EPM) test (h) and tail suspension test (TST) (i). All data are expressed as the mean value ± *SEM*. **p < 0.01, ***p < 0.0001, two‐way ANOVA, followed by Bonferroni correction for multiple comparisons

To define the contribution of Cdc42 signaling to the behavioral impairment in the parkinsonian state, mice injected with viruses (Cdc42‐ca, Cdc42‐dn, LV‐EGFP) were subjected to behavioral tests (locomotor ability (pole test, overall rotarod performance (ORP)), cognitive function (novel object recognition (NOR) task and Y‐maze), and emotional behavior (elevated plus‐maze (EPM) and tail suspension test (TST))) after saline or MPTP treatments (Figure 2a).

Motor defects, such as slowness of movement and abnormalities of coordination, are well‐known characteristics of PD (Ascherio & Schwarzschild, 2016). We found that MPTP‐treated mice showed a shortened duration time on the rotating rod (Figure 2b). These results suggest that motor coordination and balance are impaired after MPTP treatment. Although overexpression of Cdc42‐dn did not exacerbate motor deficits in ORP, overexpression of Cdc42‐ca reversed MPTP‐induced motor coordination defects (Figure 2b). We further found that MPTP‐treated mice showed a prolonged latency to turn around and land on the ground in the pole test (Figure 2c,d), indicating that sensorimotor function is impaired in parkinsonian mice. Overexpression of Cdc42‐ca partially rescued the deficits in the pole test. Overexpression of Cdc42‐dn alone impaired sensorimotor function, similar to MPTP treatment, but it did not exacerbate MPTP treatment‐induced defects in sensorimotor function (Figure 2b–d).

Cognitive impairment is the most common nonmotor symptom in PD (Morgan et al., 2015). We thus measured spatial working memory and discrimination of recognition memory by the Y‐maze test and the novel objective recognition (NOR) test, respectively. As shown in Figure 2e,f, spatial working memory was not impaired after MPTP treatment. However, in the NOR test, mice treated with MPTP or Cdc42‐dn showed a decrease in novel object exploration, and the MPTP‐induced decrease was partially reversed by Cdc42‐ca (Figure 2g). In addition, we measured anxiety and depression by elevated plus maze (EPM) and tail suspension test (TST). We found that MPTP treatment had no effect on the time or entries in the open arm of the EPM (Figure 2h) or the immobility time in the TST (Figure 2i), suggesting that anxiety and depression were not affected by MPTP treatment. Taken together, these results suggest that Cdc42 is involved in manipulating motor coordination and recognition memory in parkinsonian mice.

### 2.3 Dopamine D2R is involved in the regulation of Cdc42 activity in parkinsonian mice

**FIGURE 3:**
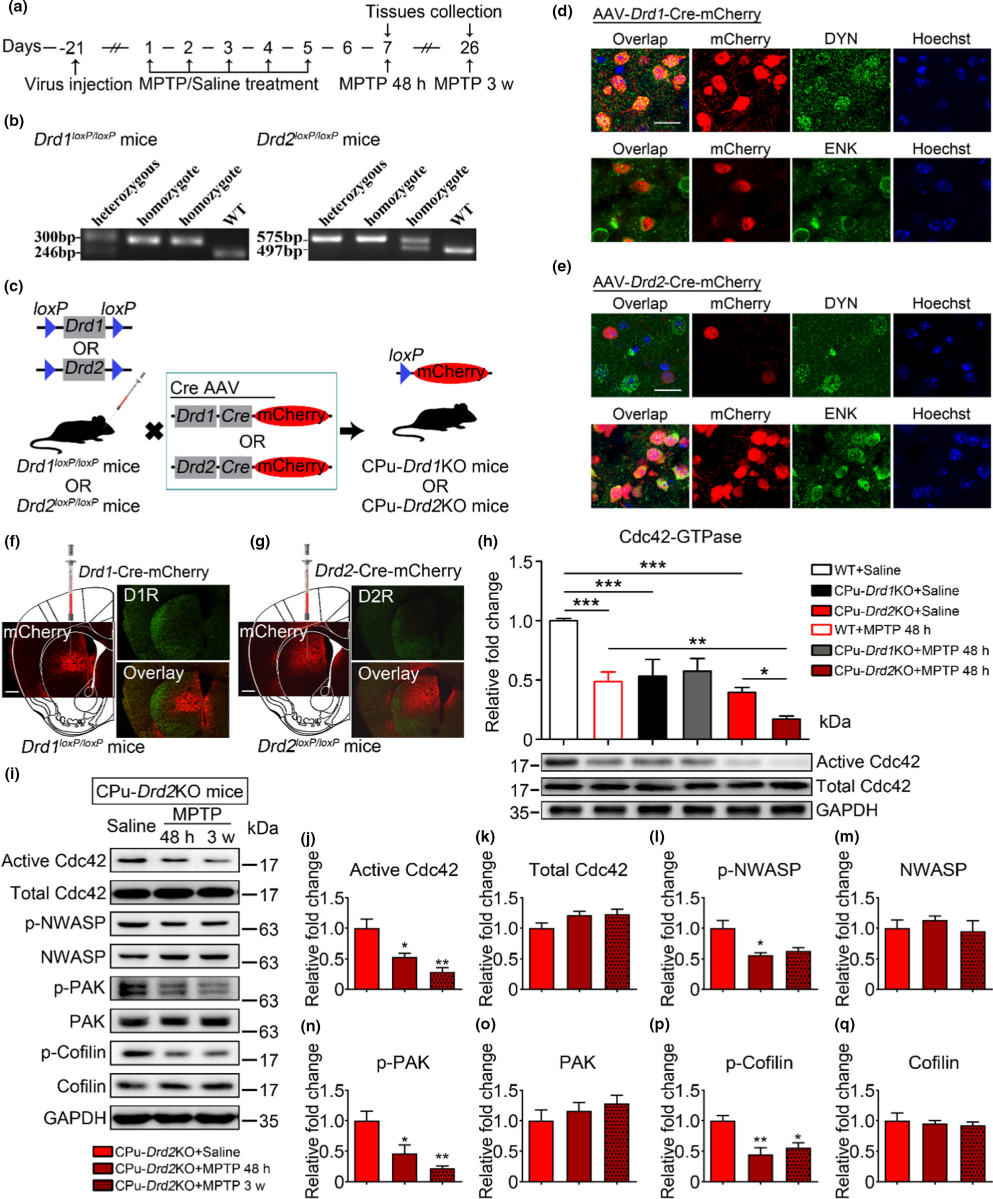
Conditional knockout of dopamine D2R in the CPu decreases Cdc42 activity in MPTP‐treated mice. (a) Timeline for successive intraperitoneal injection of MPTP or saline. (b) Polymerase chain reaction identification of *Drd1^loxP^* ^/^ *^loxP^* mice and *Drd2^loxP^* ^/^ *^loxP^* mice. (c) Schematic diagram of the construction of CPu‐*Drd1*KO mice and CPu‐*Drd2*KO mice by injecting adeno‐associated viruses (AAVs) expressing Cre recombinase tagged with mCherry driven by the dopamine D1 receptor gene (*Drd1*) promoter (*Drd1*‐Cre) or dopamine D2 receptor gene (*Drd2*) promoter (*Drd2*‐Cre) into *Drd1^loxP^* ^/^ *^loxP^* mice or *Drd2^loxP^* ^/^ *^loxP^* mice, respectively. (d) Representative images showing colocalization of *Drd1*‐Cre virus (red), dynophin (DYN) immunoreactivity (green, DYN), and hoechst (blue) in *Drd1^loxP^* ^/^ *^loxP^* mice and WT mice. Scale bar = 50 μm. (e) Representative images showing colocalization of *Drd2*‐Cre virus (red), enkephalin (ENK) immunoreactivity (green, ENK), and hoechst (blue) in *Drd2^loxP^* ^/^ *^loxP^* mice and WT mice. Scale bar = 50 μm. (f and g) Immunofluorescence staining of coronal sections showed that D1R and D2R were deleted in the CPu from CPu‐*Drd1*KO (f) and CPu‐*Drd2*KO mice (g), respectively. Red, *Drd1*‐Cre or *Drd2*‐Cre; green, D2R or D2R; blue, Hoechst. Low resolution captured by microscope (5× larger NA) and high resolution captured by laser confocal microscope (63× oil NA), Scale bars 500 μm (left), and 20 μm (right). (h) Representative western blots (bottom) and statistical analyses (top) assessing the level of Cdc42 activity in the CPu from CPu‐*Drd1*KO mice and CPu‐*Drd2*KO mice under saline and MPTP conditions. The activity of Cdc42 in CPu‐*Drd1*KO mice and CPu‐*Drd2*KO mice was decreased under saline conditions, while the activity of Cdc42 in CPu‐*Drd2*KO mice was further reduced after MPTP treatment. Total Cdc42 in the CPu was unchanged under the same conditions at the same time point (*n* = 5–7 mice/group). (i–q) Representative western blots (i) and statistical analyses (right) assessing the level of Cdc42 activity in the CPu from CPu‐*Drd2*KO mice 48 h and 3 weeks after the final injection of MPTP. The activity of Cdc42 (j) and the phosphorylated forms of N‐WASP (l), PAK (n), and Cofilin (p) were decreased in the CPu‐*Drd2*KO mice 48 h and 3 weeks after the final injection of MPTP. Total Cdc42 (k), N‐WASP (m), PAK (o), and Cofilin (q) were unaltered by MPTP at the indicated time points (*n* = 4 mice/group). The data represent the mean ± *SEM*, and the normal saline group was set to 1 for the quantitative analysis, *p < 0.05, **p < 0.001, ***p < 0.0001, two‐way ANOVA (h), followed by Bonferroni correction for multiple comparisons and one‐way ANOVA (j–q), followed by Bonferroni correction for multiple comparisons

Because both dopamine D1R and D2R signaling in the dorsal striatum play important roles in regulating behavior (Suarez et al., 2016), we examined whether the decreased Cdc42 activity in MSNs in parkinsonian mice was regulated by dopamine D1R and/or D2R. Conditional D1R and D2R knockout mice were generated by bilateral injection of adeno‐associated viruses (AAVs) expressing Cre recombinase tagged with mCherry driven by the dopamine D1R gene (*Drd1*) promoter (*Drd1*‐Cre) and dopamine D2R gene (*Drd2)* promoter (*Drd2*‐Cre) into the CPu of *Drd1* ^loxp/loxp^ and *Drd2* ^loxp/loxp^ mice, respectively. Conditional D1R knockout (CPu‐*Drd1*KO) and D2R knockout (CPu‐*Drd2*KO) mice were intraperitoneally injected with MPTP, and brain tissues were collected (Figure 3a–c). Assessment of DYN and ENK‐immunostained sections from CPu‐*Drd1*KO and CPu‐*Drd2*KO mice reveals that, as expected, *Drd1*‐Cre virus (red) in the CPu is immunoreactive for DYN (green) but little for ENK (green) (Figure 3d). In contrast, *Drd2*‐Cre virus (red) is immunoreactive for ENK (green) but is almost devoid of DYN (green) (Figure 3e). Moreover, the colocalization of *Drd1*‐Cre with dopamine D1R confirmed the absence of D1R in the virus‐infected CPu of the *Drd1* ^loxp/loxp^ mice, and the colocalization of *Drd2*‐Cre with dopamine D2R confirmed the absence of D2R in the virus‐infected CPu of the *Drd2* ^loxp/loxp^ mice (Figure 3f,g). Besides, infusion of AAV‐*Drd2*‐Cre‐mCherry viral vectors in the CPu resulted in mCherry expression (red) with no or little expression in cholinergic interneurons (green) (Figure S3). These results suggest that the *Drd1*‐Cre‐ and *Drd2*‐Cre‐mediated gene knockout approach is highly efficient.

We next explored whether knockout of D1R and D2R in the CPu alters the activity of Cdc42 in saline‐ or MPTP‐treated mice. We found that Cdc42 activity was significantly reduced after conditional knockout of D1R and D2R in the CPu under saline conditions (Figure 3h). Additionally, Cdc42 activity under parkinsonian state was further reduced in CPu‐*Drd2*KO mice 48 h after the final injection of MPTP (Figure 3i–q). Consistently, the reduction of Cdc42 signaling was detected in CPu‐*Drd2*KO mice 3 weeks after the final injection of MPTP (Figure 3i–q). These results suggest that Cdc42 signaling regulated by D2R in the CPu plays an important role in parkinsonian mice.

### 2.4 Increased Cdc42 activity rescues abnormal spine morphology and behavioral defects in CPu‐*Drd2*KO mice

**FIGURE 4:**
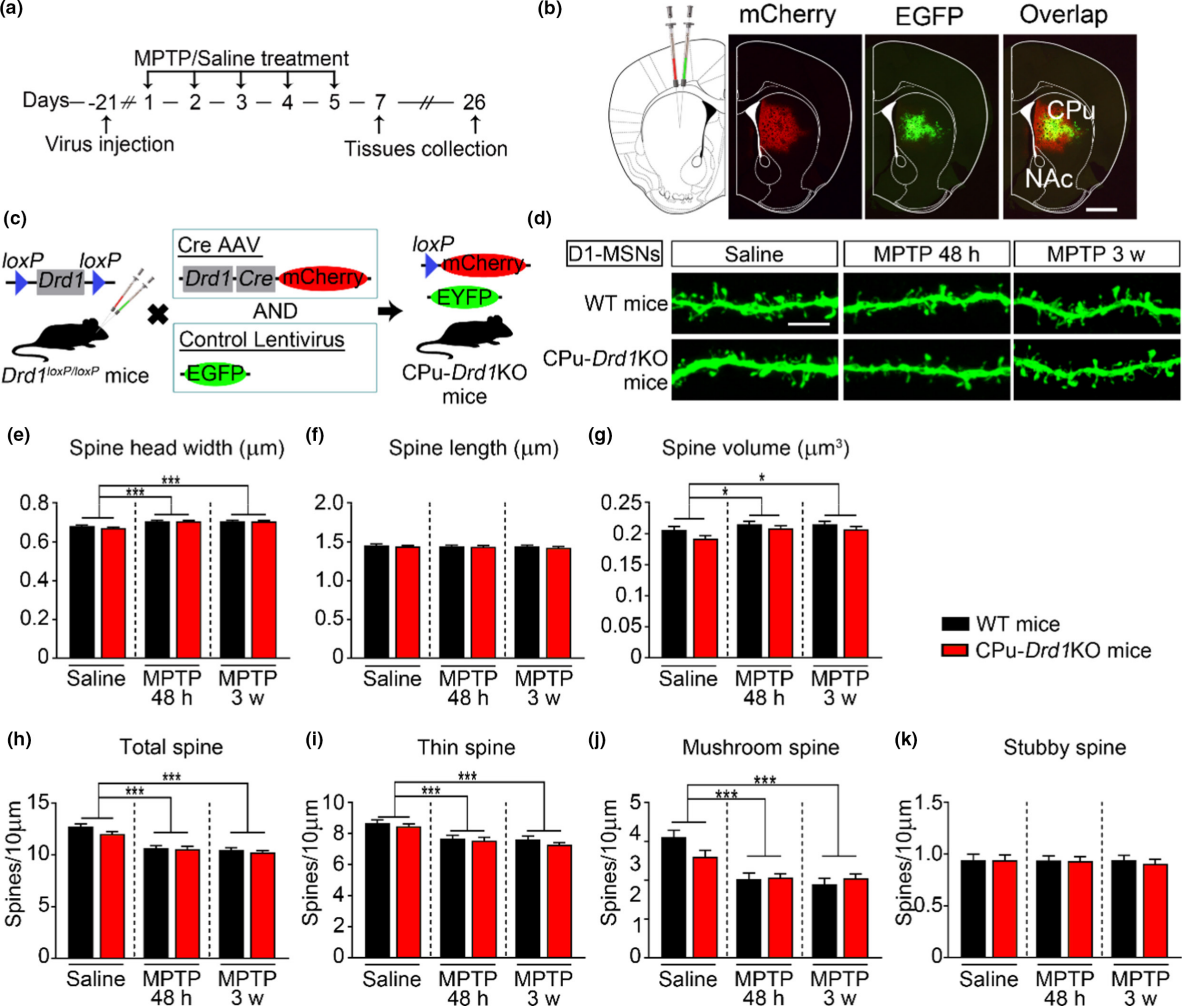
Conditional knockout of dopamine D1R in the CPu has no effect on dMSNs spine density. (a) Timeline for spine analysis for the mice in each group. (b) Representative low‐magnification image (right) of captured CPu areas with virus injection and double immunostaining showing that GFP (green) and mCherry (red) were coexpressed. Scale bar, 1 mm. (c) A schematic diagram of the coinjected *Drd1*‐Cre recombinase virus or *Drd1*‐mCherry and LV‐EGFP in *Drd1^loxp^* ^/^ *^loxp^* mice. (d) Representative image of dendritic spines of dMSNs at both 48 h and 3 weeks. Scale bar, 5 μm. (e–h) Statistical analysis of average spine head width (e), spine length (f), spine volume (g), and total spine density (h) from virus‐transfected dMSNs in the CPu at both 48 h and 3 weeks. (i–k) Statistical analysis of dendritic spine density of dMSNs, including the thin (i), mushroom (j), and stubby (k) spine subtypes at both 48 h and 3 weeks. The data represent the mean ± *SEM*. *p < 0.05, **p < 0.001, ***p < 0.0001, two‐way ANOVA, followed by Bonferroni correction for multiple comparisons

Dramatic loss of dendritic spines on MSNs is one of the most consistent biological events in animal models of PD and patients with PD. To explore whether deletion of D1R affects dendritic spines of dMSNs under parkinsonian state, CPu‐*Drd1*KO mice were bilaterally injected with LV‐EGFP into the CPu, and brain tissues were collected after intraperitoneal MPTP injection (Figure 4a,b,c). The mice that received the virus injection were recovered from the surgery for 3 weeks before spine analyses were performed (Figure 4a). Consistently, we found that MPTP treatment significantly increased spine head width (Figure 4d,e) and volume (Figure 4d,g) without changes in the average spine length (Figure 4d,f) in dMSNs. We further found that MPTP treatment reduced the total spine density (Figure 4d,h) and that this decrease was primarily due to the loss of thin (Figure 4d,i) and mushroom spines (Figure 4d,j) rather than stubby spines (Figure 4d,k) in dMSNs. However, conditional knockout of D1R in dMSNs had no effect on spine head width (Figure 4d,e), average spine length (Figure 4d,f), spine volume (Figure 4d,g) or spine density (Figure 4d,h–k) with or without MPTP treatment. Therefore, our results indicated that deletion of D1Rs in the CPu did not affect the dendritic spines of dMSNs in parkinsonian mice.

**FIGURE 5:**
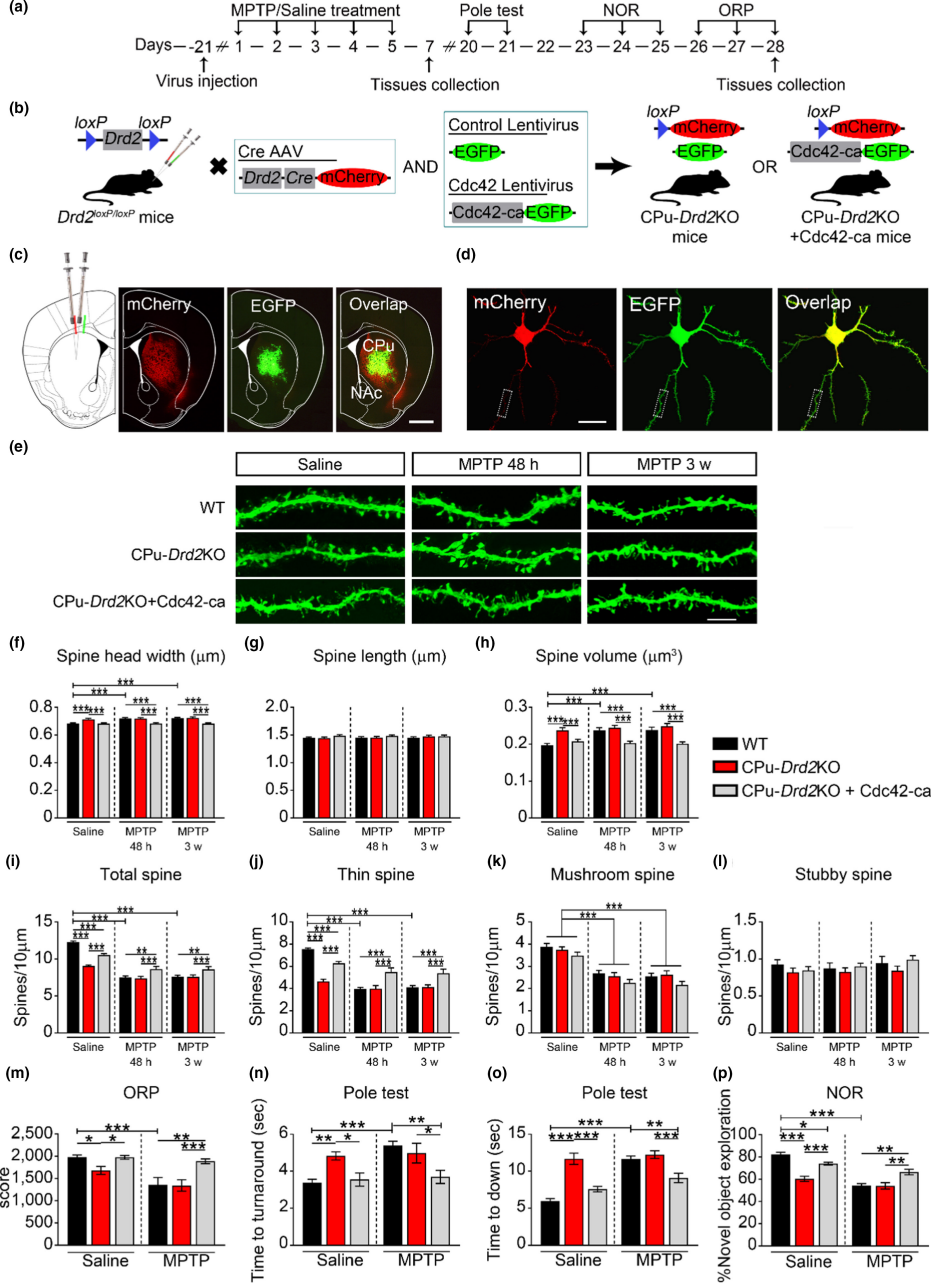
Increased Cdc42 activity rescues spine and behavioral defects in CPu‐*Drd2*KO mice after MPTP treatment. (a) Timeline for analysis of the spine structure and behavior of the mice in each group. (b) A schematic diagram of the coinjected *Drd2*‐Cre recombinase virus and Cdc42‐ca or LV‐EGFP in *Drd2^loxP^* ^/^ *^loxP^* mice. (c and d) Representative low‐magnification (c) and confocal (d) photomicrographs of captured CPu areas with virus injection and double immunostaining showing that GFP (green) and mCherry (red) were coexpressed in MSNs. Scale bar, 1 mm (c) and 20 μm (d). (e) Representative image of dendritic spines of iMSNs at both 48 h and 3 weeks. Scale bar, 5 μm. (f‐i) Statistical analysis of average spine head width (f), spine length (g), spine volume (h), and total spine density (i) from virus‐transfected iMSNs in the CPu at both 48 h and 3 weeks. (j–l) Statistical analysis of dendritic spine density of iMSNs, including the thin (j), mushroom (k), and stubby (l) spine subtypes at both 48 h and 3 weeks. (m–p) Quantification of behavioral parameters from mice in each group. Motor coordination was assessed by performance score in ORP (m) and latency to turn around (n) and land on the ground (o) in the pole test. Cognitive function was measured by the percentage of time spent exploring the novel object in the NOR task (p). The data represent the mean ± *SEM*. *p < 0.05, **p < 0.001, ***p < 0.0001, two‐way ANOVA, followed by Bonferroni correction for multiple comparisons

Subsequently, we investigated spine morphology and density in iMSNs in CPu‐*Drd2*KO mice with parkinsonian syndrome. LV‐EGFP was injected into the mouse CPu, and then the animals were subjected to behavioral tests and spine analyses (Figure 5a,b). Only dendritic spines from MSNs infected with both *Drd2*‐Cre and LV‐EGFP were selected for spine analysis (Figure 5c,d). Similar to our previous findings, iMSNs exhibited dendritic spine abnormalities under parkinsonian state (Figure 5e). Conditional knockout of D2R in iMSNs increased spine head width and volume (Figure 5e,f,h) without changing the average spine length (Figure 5e,g). Moreover, conditional knockout of D2R significantly reduced the total spine density in iMSNs (Figure 5e,i–l); this decrease was primarily due to the loss of thin spines and was further aggravated after MPTP treatment (Figure 5j). As shown in Figure 5k, conditional knockout of D2R in iMSNs had no effect on mushroom spine density. Furthermore, previous studies found that deletion or inducible ablation of D2R induced a pronounced reduction of Drd2 mRNA (>80%), with no reduction in Drd1 mRNA levels, in striatal tissue relative to the control (Lemos et al., 2016). To exclude an effect of D2R deletion on the dendritic spines of dMSNs under parkinsonian state, we next injected *Drd1*‐EGFP into the CPu of CPu‐*Drd2*KO mice (Figure S6). We found that conditional knockout of D2R had no effect on spine density in dMSNs, which only suffered dendritic spine loss after treated with MPTP (Figure S6). Taken together, these results suggest that deletion of D2R in the CPu merely exhibited aberrant spine morphology and density in iMSNs, but not dMSNs under parkinsonian state.

Next, we investigated whether restoration of Cdc42 activity in the CPu‐*Drd2*KO mice with parkinsonian syndrome could correct aberrant spine morphology and density in iMSNs. Cdc42‐ca or LV‐EGFP was injected into the CPu of D2R knockout mice. Overexpression of Cdc42‐ca corrected the enlargement of spine heads and the increase in spine volume induced by the loss of D2R in iMSNs under saline and MPTP conditions (Figure 5f,h), and partially rescued the reduction in total and thin spine density (Figure 5i,j) in iMSNs from CPu‐*Drd2*KO mice.

To test whether Cdc42‐ca could also rescue the impaired motor coordination and recognition memory induced by conditional D2 knockout in the CPu, mice co‐injected with *Drd2*‐Cre and either Cdc42‐ca or LV‐EGFP were subjected to behavioral tests after saline or MPTP treatment (Figure 5a). We observed that the CPu‐*Drd2*KO mice showed a shortened duration on the rotating rod (Figure 5m), suggesting that motor coordination and balance were impaired after conditional knockout of D2R. In addition, overexpression of Cdc42‐ca reversed D2R knockout‐induced motor coordination defects (Figure 5m). We further found that the CPu‐*Drd2*KO mice showed prolonged latency to turn around and land on the ground in the pole test (Figure 5n,o), indicating that sensorimotor function is impaired after conditional knockout of D2R. Moreover, overexpression of Cdc42‐ca rescued such deficits in the pole test (Figure 5n,o). Furthermore, in the NOR test, CPu‐*Drd2*KO mice showed decreased novel object exploration after MPTP treatment, and this decrease was partially reversed by Cdc42‐ca (Figure 5p).

Collectively, these results suggest that the inhibition of Cdc42 activity in D2R knockout mice is necessary for MPTP‐induced spine abnormalities as well as motor and cognitive deficits.

### 2.5 Deletion of Cdc42 from D2R‐positive neurons induced spine and behavioral defects, similar to that in parkinsonian mice

**FIGURE 6:**
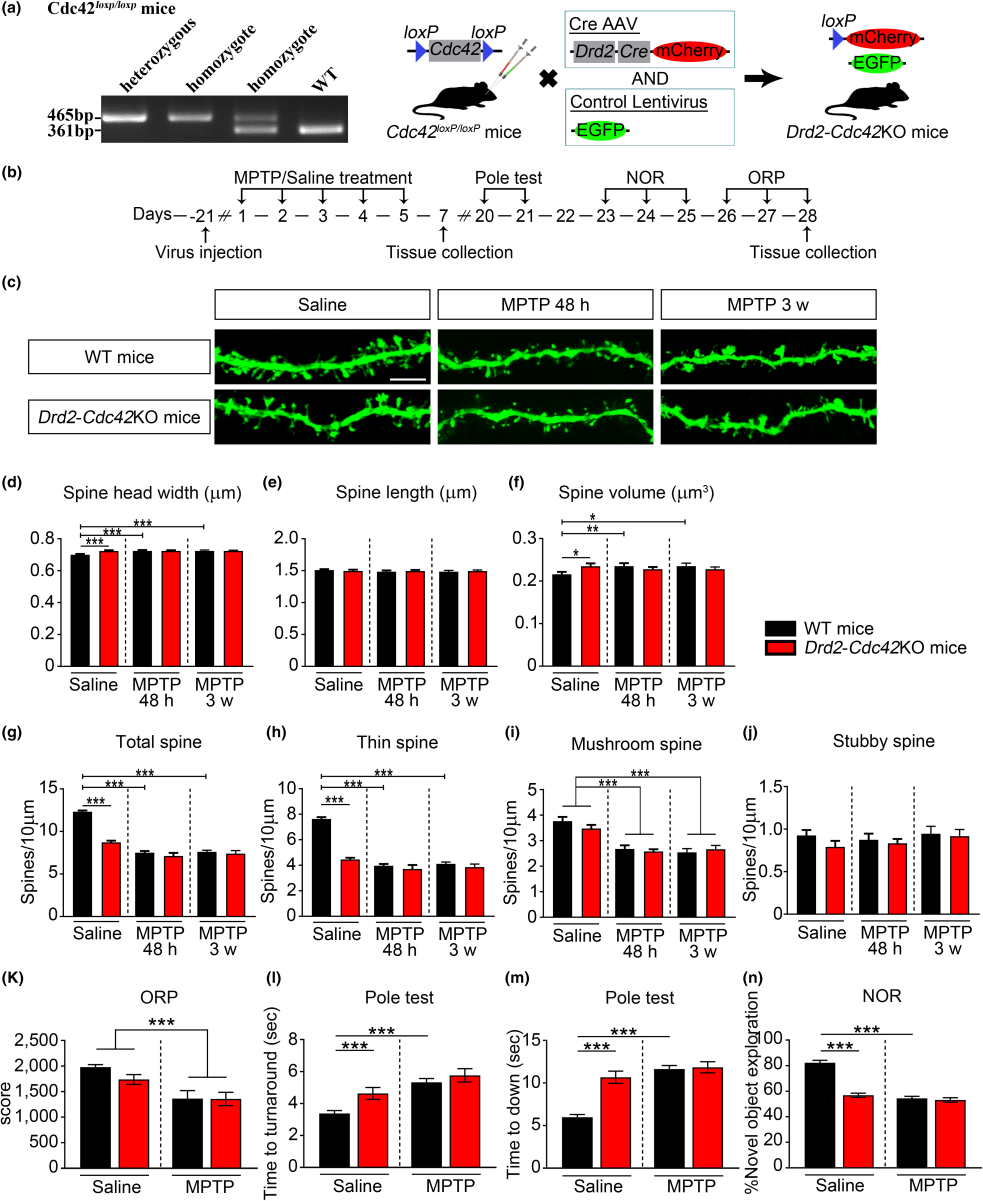
Deletion of *Cdc42* in D2R‐positive neurons of the CPu leads to spine loss and abnormal behavior. (a) Polymerase chain reaction identification of *Cdc42^loxP^* ^/^ *^loxP^* mice and schematic diagram of generating *Drd2*‐*Cdc42*KO mice by injecting *Drd2*‐Cre into *Cdc42^loxP^* ^/^ *^loxP^* mice. (b) Timeline for analysis of the spine structure and behavior of the mice in each group. (c) Representative image of dendritic spines of MSNs in *Drd2*‐*Cdc42*KO mice treated with saline and MPTP at both 48 h and 3 weeks. Scale bars, 5 μm. (d–g) Statistical analysis of average spine head width (d), spine length (e), spine volume (f), and total spine density (g) from virus‐transfected iMSNs in the CPu at both 48 h and 3 weeks. (h–j) Statistical analysis of dendritic spine density of iMSNs, including the thin (h), mushroom (i), and stubby (j) spine subtypes at both 48 h and 3 weeks. (k‐n) Quantification of behavioral parameters from mice in each group. Motor coordination was assessed by the performance score in ORP (k) and latency to turn around (l) and land on the ground (m) in the pole test. Cognitive function was measured by the percentage of time spent exploring the novel object in the NOR task (n). All data are expressed as the mean value ± *SEM*. *p < 0.05, **p < 0.001, ***p < 0.0001, two‐way ANOVA, followed by Bonferroni correction for multiple comparisons

To substantiate the role of Cdc42 in D2R‐positive neurons in the spine structure and behavioral characteristics of PD, we generated conditional Cdc42 knockout (*Drd2*‐Cdc42KO) mice, by co‐injection of *Drd2*‐Cre and LV‐EGFP into the CPu of Cdc42^loxp/loxp^ mice (Figure 6a). As expected, a reduction of Cdc42 was observed in the striatum of *Drd2*‐Cdc42KO mice compared to WT mice (Figure S4). Mice were allowed to recover for 3 weeks to fully express the virus before the spine analysis and behavioral tests (Figure 6b). WT mice injected with *Drd2*‐Cre and LV‐EGFP were used as controls. We found that conditional knockout of Cdc42 from D2R‐positive neurons in the CPu significantly increased spine head width and volume (Figure 6c,d,f) without significantly changing the average spine length (Figure 6c,e). The conditional knockout of Cdc42 from D2R‐positive neurons in the CPu reduced the total spine density (Figure 6c,g), and this decrease was mainly due to the loss of thin (Figure 6c,h) rather than mushroom (Figure 6c,i) or stubby spine density (Figure 6c,j). Furthermore, *Drd2*‐Cdc42KO mice exhibited a shortened duration on the rotating rod (Figure 6k), suggesting that motor coordination and balance were impaired after conditional knockout of Cdc42 in D2R‐positive neurons. We further found that *Drd2*‐Cdc42KO mice showed prolonged latency to turn around and land on the ground in the pole test (Figure 6l,m), indicating that conditional knockout of Cdc42 from D2R‐positive neurons is sufficient to impair sensorimotor function. In the NOR test, conditional Cdc42 knockout mice showed a decrease in novel object exploration (Figure 6n). In summary, these results reveal that conditional Cdc42 knockout mice exhibit phenotypes highly reminiscent of those in parkinsonian mice, further supporting the role of Cdc42 in D2R‐positive neurons in spine abnormalities as well as motor and cognitive deficits in parkinsonian mice.

## 3 DISCUSSION

In this study, we found a significant reduction in Cdc42 activity in the CPu of parkinsonian mice that was necessary for spine and behavioral plasticity in parkinsonian mice. We further demonstrated that the decreased Cdc42 signaling was primarily regulated by D2R but not D1R and mediates the spine and behavioral defects in parkinsonian mice. Our findings revealed a novel mechanism in which the reduction in Cdc42 regulated by dopamine D2R contributes to the pathophysiology of parkinsonian mice, indicating that Cdc42 is an effective target for treating PD.

Cdc42 is a major determinant of spine formation and maintenance, and aberrant Cdc42 signaling in the striatum is implicated in multiple neurological diseases, such as Huntington's disease (Holbert et al., 2003). In our study, we found that Cdc42 signaling in the CPu was decreased in parkinsonian mice, which echoed previous studies reporting that Cdc42 was decreased in patients with PD (Chi et al., 2018; Habib et al., 2018; Zhang et al., 2005). However, Barcia et al. reported that total Cdc42 was increased in the nigrostriatal pathway after MPTP treatment (Barcia et al., 2012). This discrepancy may be due to the different MPTP mouse models (subchronic MPTP model in our experiments and acute MPTP model in Barcia et al.) and different dopamine concentrations in these two mouse models. In this study, we found that MPTP decreased spine density, which was accompanied by an increase in head width and spine volume. It is tempting to speculate that the increase in the head width and spine volume observed in the PD model might compensate for the decrease in dendritic spine density, as spines may need to maintain a certain synaptic strength. Moreover, we found that activation of Cdc42 in the CPu could attenuate MPTP‐induced spine abnormalities, whereas inactivation of Cdc42 in the CPu reduced the thin spine density and increased spine size (including spine head and spine volume). Therefore, it is reasonable to postulate that Cdc42 is necessary to improve synaptic strength damage after MPTP treatment. Indeed, numerous studies have indicated that decreased Cdc42 signaling contributes to decreased spine density. For example, the activation of Cdc42 increased the formation of dendritic spines in Aplysia sensory neurons (Udo et al., 2005), while the inactivation of Cdc42 reduced the dendritic spine density in vertical system neurons in Drosophila (Scott et al., 2003). In addition, a mathematical model predicted that depletion of Cdc42 slightly altered the spine volume (Rangamani et al., 2016). Together, these studies indicate that Cdc42 may be essential for spine plasticity.

In accordance with previous work showing that MPTP‐treated mice display deficits in motor coordination and cognitive function (Moriguchi et al., 2012), the MPTP‐treated mice in our research exhibited deficits in motor coordination and cognition but normal basal motor activity and affective behavior. Moreover, we found that constitutively active Cdc42 in the CPu was sufficient to attenuate MPTP‐induced motor coordination and cognitive deficits. And dominant negative Cdc42 impaired motor coordination and cognitive function, similar to MPTP treatment, and it exacerbated MPTP‐induced motor coordination deficit. These results suggest that the decrease in Cdc42 signaling is important for behavioral plasticity in parkinsonian mice. Converging evidence has shown that Cdc42 is critical for the regulation of motor coordination and cognitive behavior. The activation of Cdc42 is involved in anesthesia‐resistant memory formation (Zhang et al., 2016), and the deletion of Cdc42 impairs balance and motor coordination in mice with vestibular dysgenesis (Kaartinen et al., 2002). Additionally, knockout of Cdc42 or effector proteins of Cdc42 impaired locomotion and memory (Kim et al., 2014; Nekrasova et al., 2008). Thus, we confirmed that the downregulation of Cdc42 in the CPu was responsible for the impairment in motor coordination and cognition in parkinsonian mice. Moreover, the finding that decreased Cdc42 signaling is responsible for decreased spine density and cognitive dysfunction in schizophrenia further supports the role of Cdc42 in regulating spine and behavior (Ide & Lewis, 2010).

Accumulating evidence indicates that dopamine receptors are critical for striatal MSNs spine remodeling in neuropsychiatric diseases, such as PD (Beeler et al., 2012). Some studies report that both dMSNs and iMSNs undergo robust spine loss after dopaminergic denervation (Bello et al., 2017). Using the *Drd1* or *Drd2* promoter to distinguish dMSNs or iMSNs, respectively, in the CPu, our results demonstrated that spine abnormalities in parkinsonian mice occurred not only in dMSNs but also in iMSNs. Furthermore, conditional D1R deletion in the CPu had no effect on spine density in dMSNs, but conditional D2R deletion in the CPu significantly decreased the spine density and enlarged the spine head and volume in the iMSNs under a parkinsonian state. Consistently, genetic studies have revealed that loss of the intracellular D2R long isoform but not D1R decreases the spine density of striatal MSNs (Shioda et al., 2017). This evidence indicates that dopamine D2R is important for spine remodeling in PD.

In addition, dopamine receptors coupled to G proteins are notable for engaging a multitude of intracellular molecules to manipulate structural remodeling, conferring their psychomotor functions, by regulating Rho family GTPases (Francis et al., 2019; Gurevich et al., 2016). G proteins such as Gα_12_ have been shown to be linked to Cdc42, which is sufficient for spine remodeling (Vanni et al., 2007). In the present study, we found that Cdc42 activity was regulated by D2R, but not D1R, under a parkinsonian state. Our data are consistent with a recent high‐throughput RNA‐seq study showing that transcripts encoding Cdc42 are enriched in iMSNs of the dorsal striatum (Puighermanal et al., 2020). Furthermore, Suarez et al indicate that iMSNs are slightly affected by deletion of D1R but strongly affected by dopamine depletion (Suarez et al., 2020). These findings may explain why Cdc42 signaling is mainly regulated by D2R, but not D1R, under parkinsonism state. Interestingly, we found that Cdc42 activity in the CPu was decreased by conditional D2R deletion in the CPu and further reduced after MPTP treatment, while Cdc42 activity was decreased by conditional D1R deletion in the CPu but was unaffected by MPTP treatment, suggesting that Cdc42 is primarily controlled by D2R in parkinsonian mice. Our investigations are reminiscent of our previous work in drug addiction showing that both dopamine D1R and D2R activate Cdc42 signaling to manipulate spine plasticity concomitant with methamphetamine‐induced conditioned place preference (Tu et al., 2019). However, evidence suggests that D1R activation occurs with exposure to large amounts of dopamine because of its low affinity to dopamine, and D2Rs may be activated by relatively lower levels of dopamine due to its high affinity to dopamine (Volkow et al., 2013). Thus, Cdc42 may be regulated by dopamine D2R but not D1R under the lower levels of dopamine found in PD.

D2R in the striatum is essential for orchestrating motor output and is abundant on numerous types of neurons, such as iMSNs, cholinergic interneurons (CINs) and GABA interneurons (Lemos et al., 2016). At this time, it is unclear in which cell types D2R lost in the CPu is playing a role. Loss of D2Rs in interneurons iMSNs may be also responsible for spine loss. It is worth noting that D James Surmeier and his colleagues have uncovered that cholinergic signaling is essential for spine pruning in models of Parkinson's disease (Shen et al., 2007). Compared with MSNs, striatal CINs only account for 1–3% of the total striatal neuronal population (Cai et al., 2021). However, they have previously shown to be critical for motor behavior and striatally based associative learning (Cai et al., 2021). But we found the AAV‐*Drd2*‐Cre‐mCherry virus can hardly infect cholinergic interneurons, indicating the deletion of D2R mainly occurred in iMSNs in our study. Furthermore, conditional knockout of Cdc42 from D2R‐positive neurons in the CPu mimicked an array of Parkinson‐like phenotypes including spine abnormalities and behavioral deficits, suggesting that Cdc42, likely in iMSNs, is implicated in modulating spine and behavioral plasticity in parkinsonian mice. In addition, our data are consistent with a recent high‐throughput RNA‐seq study showing that transcripts encoding Cdc42 are enriched in iMSNs of the dorsal striatum (Puighermanal et al., 2020). Thus, we speculated that iMSNs could be the main cell types regulating the striatal behaviors in our study. Limited by the methods and the mouse strains currently have, we can only locate our target cells in D2R‐positive neurons and D1R‐positive neurons. In addition, we do not have the Chat‐Cre mice or AdorA2A mice, at this point, to directly determine if loss of D2Rs in interneurons is responsible for spine loss. We are constantly enriching our experimental level and mouse strains and carry out further verification on what cell type Cdc42 plays function in the future.

## 4 CONCLUSION

In summary, our current observations suggest a novel mechanism in which Cdc42 signaling in the CPu, regulated by dopamine D2R, contributes to deficits in spine abnormities and motor coordination and cognition, characteristic of Parkinson's disease. Deletion of D2R is widely reported to be responsible for Parkinson‐like behavior, such as deficits in motor coordination and cognitive function. Over the years, different D2R agonists have been widely applied in clinical practice to treat Parkinsonism. However, the side effects of these D2R agonists more or less impair quality of life in patients with PD. Combined with our findings that Cdc42 is closely related to D2R and is essential for ameliorating deficits in spine morphology and behavior performance in parkinsonian mice, it is tempting to speculate that Cdc42 is a promising therapeutic target for improving motor and cognitive deficits in Parkinson's disease.

## 5 EXPERIMENTAL PROCEDURES

### 5.1 Animals

The animal and MPTP administration details are fully described in Supplementary material.

### 5.2 Viral constructs and stereotaxic injection

Lentiviruses containing constitutively active (Cdc42‐ca) and dominant negative Cdc42 (Cdc42‐dn) driven by the CMV promoter were combined with EGFP and constructed by Obio Technology Corp., Ltd. Control lentivirus expressed EGFP alone (Tu et al., 2019). Recombinant adeno‐associated virus serotype 2/9 (AAV 2/9) expressing mCherry/EYFP/EGFP alone or in combination with the Cre enzyme driven by the dopamine D1 receptor gene promoter (*Drd1*) or dopamine D2 receptor gene promoter (*Drd2*) was constructed by BrainVTA (Wuhan, China) (Tu et al., 2019; Zhao et al., 2019). All viruses’ constructs are listed in Table S3. According to a previous study (Zhao et al., 2019), mice were anesthetized with 50 mg/kg pentobarbital sodium and positioned in a stereotaxic instrument. The skull was exposed, and holes were drilled above the CPu using standard procedures. The injection coordinates of the CPu were 0.7 mm rostral, 1.6 mm lateral, and 3.3 mm ventral. The mice received bilateral injections at a low rate (0.1 µl/min) by a Hamilton 0.5 μl syringe and a 7000 SER, 32 ga. Cole‐Parmer infusion pump. The injector needle was retained for no <5 min after the infusion to promote full diffusion and then slowly withdrawn to prevent backflow. Mice recovered for 3 weeks after the surgery to ensure viral expression.

### 5.3 Immunohistochemistry and immunofluorescence staining

Details were described in Supplementary Material. The dilutions of each antibody are listed in Table S4.

### 5.4 Confocal imaging and spine acquisition

Details were described in Supplementary Material.

### 5.5 Spine reconstruction and analysis

A semiautomated software program, that is, NeuronStudio, was used to process the morphological features of dendritic spines (Rodriguez et al., 2006) (http://research.mssm.edu/cnic/tools‐ns.html). Details were described in Supplementary Material.

### 5.6 Pull‐down assay and Western blotting

The pull‐down assay was performed according to the manufacturer's protocol, and western blotting was performed. Details were described in Supplementary Material. The dilutions of each antibody are listed in Table S5.

### 5.7 Behavioral assays

Details on behavioral assays were described in Supplementary material.

### 5.8 Statistical analysis

Statistical analysis was performed in SPSS 19.0, and the pictures in each figure were arranged by Prism (GraphPad). Details were described in Supplementary Material.

## CONFLICT OF INTEREST

The authors report no competing financial or other interests.

## AUTHORS' CONTRIBUTIONS

Lu Z., Lin Z, and NL. were responsible for the overall study design and drafted the paper with the help of other authors. LY, JLZ, YTL, YSY, TX, HFZ, YW, BX, JH, and SFQ conducted experiments. FKG and YJ provided technical support. LY and JLZ interpreted the data and prepared all the figures. All authors read and approved the final manuscript.

## Supporting information

Supplementary Material

Fig S1‐S6

Table S1‐S17

## Data Availability

All data generated or used during the study are available from the corresponding author on request.
