# Supplementary material for: Regulation of Cdc42 signaling by the dopamine D2 receptor in a mouse model of Parkinson’s disease
Source: Aging Cell. 2022 Apr 12;21(5):e13588. doi: 10.1111/acel.13588 (PMC9124300; doi:10.1111/acel.13588)
Supplement: Supplementary file 1 — Supplementary Material [file ACEL-21-e13588-s003.docx]

**Supplementary material**

**Animals and MPTP administration**

All mice in this study were male due to the sex disparities in Parkinson's disease (Sharma et al., 2020), and the animal details are fully described in Table S1. The mice weighed 22-26 g and were 8-10 weeks of age at the start of testing. The mice were randomly group-housed in plastic cages in a humidity and temperature-controlled room (20-22 °C) under a 12/12-h light/dark cycle with ad libitum access to food and water. For the genotyping of the mice, tail tissues were digested overnight, and DNA was extracted. The genotyping primers and PCR schemes are listed in Table S2. All of the animals were handled according to approved institutional animal care and use committee (IACUC) protocols (#L2017246) of Southern Medical University. Every effort was made to improve animal welfare, and the number of animals used, and any suffering caused, were all kept to a minimum.

To duplicate a stable mouse model of PD, we used a subchronic MPTP regimen as described previously (Jackson-Lewis & Przedborski, 2007). Briefly, for the MPTP-treated mice, an intraperitoneal (i.p.) injection of 30 mg/kg (free base) MPTP (Sigma-Aldrich, St. Louis, MO, USA) was administered daily for 5 consecutive days; the saline-treated mice received 0.9% sterile saline injections.

**Cannula implantation and microinjection**

Mice were anesthetized with 50 mg/kg pentobarbital sodium and positioned in a stereotaxic instrument. Then, mice were bilaterally implanted with 26-gauge guide cannulae (RWD Life Science Co., Ltd.) located 0.5 mm above the CPu at the following coordinates: anteroposterior (AP), +0.9 mm; mediolateral (ML), ±1.5 mm; dorsoventral (DV), −2.8 mm. The cannulae were fixed to the skull with dental cement, and a needle was inserted into the cannula to prevent obstruction. Mice were allowed a week recover from surgery before the subsequent behavioral tests. The 28-gauge injection injector protruding 0.5 mm from the guide cannula was connected to Hamilton syringes using polyethylene tubing. Quinpirole (0.5 μg/μl, 0.8 μl/side) or vehicle (0.8 μl/side) was infused into the bilateral CPu at a rate of 0.1 μl/min. The doses of drugs are based on previous studies with slightly modified (Guo et al., 2021; Kiessling, Lanza, Feinberg, & Bishop, 2020; Nasehi, Hasanvand, Khakpai, & Zarrindast, 2019). After infusion, the injector remained in the cannula for 5 min to allow time for the drug diffusion. The mice were returned to home cages for another 20-30 min prior to engaging in the behavioral tests. The correct cannula placement was verified at the end of the tests using Nissl staining.

**Immunohistochemistry and immunofluorescence staining**

The mice were anesthetized with 50 mg/kg pentobarbital sodium and perfused intracardially with 4% paraformaldehyde (PFA). The brains were removed, postfixed overnight in 4% PFA, dehydrated in 30% sucrose at 4°C, and cut into frozen coronal sections at 30 µm by a cryostat microtome (Leica, VT1200, Germany). The free-floating sections were collected and immersed at 4°C for cryoprotection. For the immunofluorescence analyses, the sections were incubated with the primary antibody at 4°C overnight, followed by incubation with the secondary antibody for 2 h at room temperature; subsequently, the sections were mounted on gelatin-coated slides to capture the images. The dilutions of each antibody are listed in Table S4.

**Confocal imaging and spine acquisition**

Morphological features such as the size, shape and density of dendritic spines play important roles in neurological diseases. We analyzed MSNs spine density on dendritic segments (diameters <1 μm) of secondary dendrites at least 50 μm in length, as previously described (Zhao et al., 2019). Only the green channel was used for spiny dendrite capture to avoid measurement bias caused by different fluorescent signals. The images of the segments were acquired under 63× oil magnification with 0.044 µm × 0.044 µm resolution at 0.4 μm z-steps by confocal microscopy (Zeiss LSM 700 Carl Zeiss Microscopy, USA). Before each image was captured, confocal parameters were set to exhibit the morphology of the dendritic spines as clearly as possible under the microscope.

**Spine reconstruction and analysis**

A semiautomated software program, i.e., NeuronStudio, was used to process the morphological features of dendritic spines (Rodriguez, Ehlenberger, Hof, & Wearne, 2006) (<http://research.mssm.edu/cnic/tools-ns.html>). Furthermore, NeuronStudio categorized spines into the following three subtypes according to the parameters of the spine head and spine neck: mushroom spine, which has a large head and narrow neck; thin spine, which has a thin and long neck and a small head; and stubby spine, which does not have a neck. Researchers blinded to the mouse groups judged all categories of dendritic spines and corrected any erroneous assignments.

**Pull-down assay and Western blotting**

The pull-down assay was performed according to the manufacturer's protocol, and western blotting was performed as previously described (Xu et al., 2018; Zhao et al., 2019). The mice were anesthetized and decapitated, and the mouse brains were quickly removed and immersed in buffer solution at 4°C. The CPu was quickly isolated by gross dissection, and the samples were lysed with buffer containing Mg2+ (MLB; 25 mM HEPES, pH 7.5, 150 mM NaCl, 1% Triton X-100, 0.25% sodium deoxycholate, 10% glycerol, 25 mM NaF, 10 mM MgCl2, 1 mM EDTA, 1 mM sodium orthovanadate, 10 μg/ml leupeptin, and 10 μg/ml aprotinin). The protein concentrations were measured by the Bradford method. The lysates were divided into the following two parts: one part was used to isolate the active form of Cdc42 (active Cdc42) for the pull-down assays, while the other part was used to detect the total proteins by western blotting. The proteins were separated by 10% SDS-PAGE and transferred onto PVDF membranes by gel electrophoresis. Then, the membranes were blocked for 1 h with Tris-buffered saline containing 0.1% Tween-20 and 5% nonfat milk and successively incubated with primary and secondary antibodies diluted in blocking agent. The bands on the membranes were visualized by a chemiluminescent reagent. Loading controls were performed using antibodies against mouse GAPDH. The dilutions of each antibody are listed in Table S5.

**Behavioral assays**

Behavioral assays were used to determine the phenotype of the mice in our study. These tests were administered in a sequence from least to most stressful. All mice were handled before the behavioral tests. Rotarod performance is a reliable test to assess motor coordination and balance (Brooks & Dunnett, 2009). The two-trial Y-maze paradigm evaluates MPTP-induced impairment in spatial memory (Castonguay et al., 2018). The novel object recognition test is based on the ability of the mice to discriminate a new object from a familiar one (Lipina et al., 2016; Zhou et al., 2019). The elevated plus-maze and Tail suspension test is thought to reflect despair as a symptom of anxiety and depression. All these tests were administered in a sequence from least to most stressful (Crawley, 2008).

Overall rotarod performance (ORP)

The endurance of the mice was examined on a rotating spindle using the protocol described by Rozas et al *al* (Rozas, Guerra, & Labandeira-García, 1997; Rozas, Lopez-Martin, Guerra, & Labandeira-Garcia, 1998). Each animal was placed on a rod rotating at a low speed of 5 rpm for acclimation for 10 min. During the acclimatization period, the mice that fell were placed back on the rod. During the test, the mice were tested in 8 trials at a gradually accelerating speed from 12 rpm to 26 rpm at an interval of 2 rpm, and the latency to fall was recorded. The maximal duration of each trial and the interval between each trial were 150 seconds. The ORP of each group was calculated by plotting the average latency to fall at each speed and using the trapezoidal method described by Rozas et al. (1997) to estimate the area under the curve (Rozas et al., 1997).

Pole test

The pole test is a method used to assess MPTP-induced slowness of movement in mice following striatal dopamine depletion (Su et al., 2019). The mice were placed head-upward atop a vertical pole taped with gauze (diameter 1 cm; height 55 cm). Mice would turn head downward and descend to the floor. The test was performed on two consecutive days. The day before the test, the mice were allowed to habituate to the test by conducting five consecutive trials. On the test day, the latency for the mice to turn their head downward (time to turnaround) and the latency to descend to the floor (time to down) were recorded. The data from mice that slipped, fell or performed only parts of the pole test were not used in the analysis.

Y-maze test

The Y-maze consisted of three interconnected gray-painted arms at 120° angles. During trial one, the mice were allowed to move on the start arm and open arm for 5 min but were limited to exploring the novel arm. After a 30 min inter-trial interval, the mice were placed back and allowed to freely explore the three arms in trial two. The ratio of the time spent in the novel arm and the number of novel arm entries were calculated ((Novel/[Novel+Start+Open]) * 100%) (Zhang et al., 2011).

Novel object recognition (NOR) task

The mice were individually acclimatized to the chamber for 3 consecutive days (the acquisition phase) and were tested on the third day. During the acquisition phase, two objects of the same material were symmetrically placed in the chamber, and the mice were placed in the chamber for 5 min. On the third day, 1 h after the acquisition phase, one new object replaced one of the two old objects in the chamber, and the mice were again placed in the chamber and tracked for 5 min. After each session, both objects and the chamber were thoroughly cleaned to prevent odor cognition. Rearing at or sniffing an object at a distance of less than 1 cm or touching an object with the nose were all defined as exploration of the object. Discrimination of spatial novelty was assessed by comparing the exploration time for both objects (Liu et al., 2016).

Elevated plus-maze (EPM)

A standard plus-maze was made of plastic arms (open arms, 29 × 5 cm; closed arms, 29 × 5 × 15 cm; center, 5 × 5 cm). The mice were positioned in the center of the platform, and their behavior was tracked for 5 min. The test was carried out under dim light to encourage the mice to explore the open arms. The duration in the open arms and entries into the open arms were analyzed (Tu et al., 2019).

Tail suspension test (TST)

The mice were individually hung at a height of 20 cm above the ground using a piece of adhesive tape wrapped around the tail (2 cm). The behavior was videotaped for 5 min, and the duration of immobility was measured.

**Statistical analysis**

Statistical analysis was performed in SPSS 19.0, and the pictures in each figure were arranged by Prism (GraphPad). The number of mice in each group in our experiment is based on the estimates of previous studies. All experiments were replicated at least twice, with biological and technical replicates. The “n” for western blot and behavior analysis refers to the animals; The “n” for spine size analysis refers to dendritic spine; The “n” for spine density analysis refers to dendrite. Two-tailed independent Student's t-tests were used to compare the number of tyrosine hydroxylase (TH) neurons and TH fibers. One-way ANOVA or two-way ANOVA, followed by Bonferroni correction for multiple comparisons, was used to measure the significance of the differences in the rest of the experiments. All values are presented as the mean ± SEM. p < 0.05 was considered indicative of statistical significance (for details, see Tables S6-17).

**References**

Brooks, S. P., & Dunnett, S. B. (2009). Tests to assess motor phenotype in mice: a user's guide. *Nature reviews. Neuroscience, 10*(7), 519-529. doi:10.1038/nrn2652

Castonguay, D., Dufort-Gervais, J., Ménard, C., Chatterjee, M., Quirion, R., Bontempi, B., . . . Brouillette, J. (2018). The Tyrosine Phosphatase STEP Is Involved in Age-Related Memory Decline. *Current biology : CB, 28*(7), 1079-1089.e1074. doi:10.1016/j.cub.2018.02.047

Crawley, J. N. (2008). Behavioral phenotyping strategies for mutant mice. *Neuron, 57*(6), 809-818. doi:10.1016/j.neuron.2008.03.001

Guo, M., Xiang, T., Li, M., Sun, Y., Sun, S., Chen, D., . . . Wang, M. (2021). Effects of intrastriatal injection of the dopamine receptor agonist SKF38393 and quinpirole on locomotor behavior in hemiparkinsonism rats. *Behav Brain Res, 411*, 113339. doi:10.1016/j.bbr.2021.113339

Jackson-Lewis, V., & Przedborski, S. (2007). Protocol for the MPTP mouse model of Parkinson's disease. *Nat Protoc, 2*(1), 141-151. doi:10.1038/nprot.2006.342

Kiessling, C. Y., Lanza, K., Feinberg, E., & Bishop, C. (2020). Dopamine receptor cooperativity synergistically drives dyskinesia, motor behavior, and striatal GABA neurotransmission in hemiparkinsonian rats. *Neuropharmacology, 174*, 108138. doi:10.1016/j.neuropharm.2020.108138

Lipina, T. V., Prasad, T., Yokomaku, D., Luo, L., Connor, S. A., Kawabe, H., . . . Craig, A. M. (2016). Cognitive Deficits in Calsyntenin-2-deficient Mice Associated with Reduced GABAergic Transmission. *Neuropsychopharmacology, 41*(3), 802-810. doi:10.1038/npp.2015.206

Liu, Y., Du, S., Lv, L., Lei, B., Shi, W., Tang, Y., . . . Zhong, Y. (2016). Hippocampal Activation of Rac1 Regulates the Forgetting of Object Recognition Memory. *Current biology : CB, 26*(17), 2351-2357. doi:10.1016/j.cub.2016.06.056

Nasehi, M., Hasanvand, S., Khakpai, F., & Zarrindast, M. R. (2019). The effect of CA1 dopaminergic system on amnesia induced by harmane in mice. *Acta Neurol Belg, 119*(3), 369-377. doi:10.1007/s13760-018-0926-8

Rodriguez, A., Ehlenberger, D. B., Hof, P. R., & Wearne, S. L. (2006). Rayburst sampling, an algorithm for automated three-dimensional shape analysis from laser scanning microscopy images. *Nat Protoc, 1*(4), 2152-2161. doi:10.1038/nprot.2006.313

Rozas, G., Guerra, M. J., & Labandeira-García, J. L. (1997). An automated rotarod method for quantitative drug-free evaluation of overall motor deficits in rat models of parkinsonism. *Brain Res Brain Res Protoc, 2*(1), 75-84. doi:10.1016/s1385-299x(97)00034-2

Rozas, G., Lopez-Martin, E., Guerra, M. J., & Labandeira-Garcia, J. L. (1998). The overall rod performance test in the MPTP-treated-mouse model of Parkinsonism. *J Neurosci Methods, 83*(2), 165-175.

Sharma, A., Weber, D., Raupbach, J., Dakal, T. C., Fließbach, K., Ramirez, A., . . . Wüllner, U. (2020). Advanced glycation end products and protein carbonyl levels in plasma reveal sex-specific differences in Parkinson's and Alzheimer's disease. *Redox Biol, 34*, 101546. doi:10.1016/j.redox.2020.101546

Su, Y., Deng, M. F., Xiong, W., Xie, A. J., Guo, J., Liang, Z. H., . . . Zhu, L. Q. (2019). MicroRNA-26a/Death-Associated Protein Kinase 1 Signaling Induces Synucleinopathy and Dopaminergic Neuron Degeneration in Parkinson's Disease. *Biological psychiatry, 85*(9), 769-781. doi:10.1016/j.biopsych.2018.12.008

Tu, G., Ying, L., Ye, L., Zhao, J., Liu, N., Li, J., . . . Zhang, L. (2019). Dopamine D1 and D2 Receptors Differentially Regulate Rac1 and Cdc42 Signaling in the Nucleus Accumbens to Modulate Behavioral and Structural Plasticity After Repeated Methamphetamine Treatment. *Biological psychiatry, 86*(11), 820-835. doi:10.1016/j.biopsych.2019.03.966

Xu, S., Zhang, Y., Wang, J., Li, K., Tan, K., Liang, K., . . . Bai, X. (2018). TSC1 regulates osteoclast podosome organization and bone resorption through mTORC1 and Rac1/Cdc42. *Cell Death Differ, 25*(9), 1549-1566. doi:10.1038/s41418-017-0049-4

Zhang, J., Wang, Y., Chi, Z., Keuss, M. J., Pai, Y. M., Kang, H. C., . . . Dawson, V. L. (2011). The AAA+ ATPase Thorase regulates AMPA receptor-dependent synaptic plasticity and behavior. *Cell, 145*(2), 284-299. doi:10.1016/j.cell.2011.03.016

Zhao, J., Ying, L., Liu, Y., Liu, N., Tu, G., Zhu, M., . . . Zhang, L. (2019). Different roles of Rac1 in the acquisition and extinction of methamphetamine-associated contextual memory in the nucleus accumbens. *Theranostics, 9*(23), 7051-7071. doi:10.7150/thno.34655

Zhou, J., Chow, H.-M., Liu, Y., Wu, D., Shi, M., Li, J., . . . Zhang, J. (2019). CDK5-dependent BAG3 degradation modulates synaptic protein turnover. *Biological psychiatry*. doi:10.1016/j.biopsych.2019.11.013
