# Supplementary material for: Regulation of Cdc42 signaling by the dopamine D2 receptor in a mouse model of Parkinson’s disease
Source: Aging Cell. 2022 Apr 12;21(5):e13588. doi: 10.1111/acel.13588 (PMC9124300; doi:10.1111/acel.13588)
Supplement: Supplementary file 2 — Fig S1‐S6 [file ACEL-21-e13588-s002.docx]

**
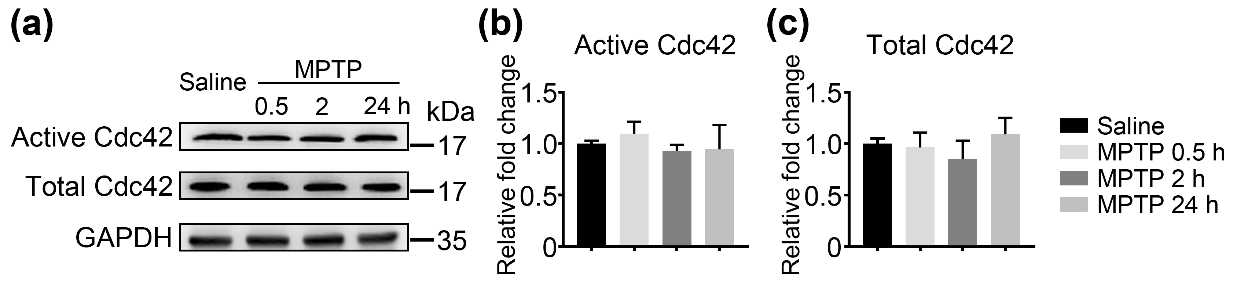
**

**Figure S1. Cdc42 activity in the CPu remained unaltered 5 h, 2 h and 24 h after the final injection of MPTP**

**(a-c)** Representative western blots (a) and statistical analyses (b and c) assessing the level of Cdc42 activity in the CPu 0.5 h, 2 h and 24 h after the final injection of MPTP. The activity of Cdc42 (b) in the CPu remained unaltered 0.5 h, 2 h and 24 h after the final injection of MPTP. Total Cdc42 (c) was not affected by MPTP at the indicated time points (n = 3 mice/group). The data represent the mean ± SEM, and the normal saline group was set to 1 for the quantitative analysis, *, p < 0.05, **, p < 0.001, ***, p < 0.0001, one-way ANOVA, followed by Bonferroni correction for multiple comparisons.


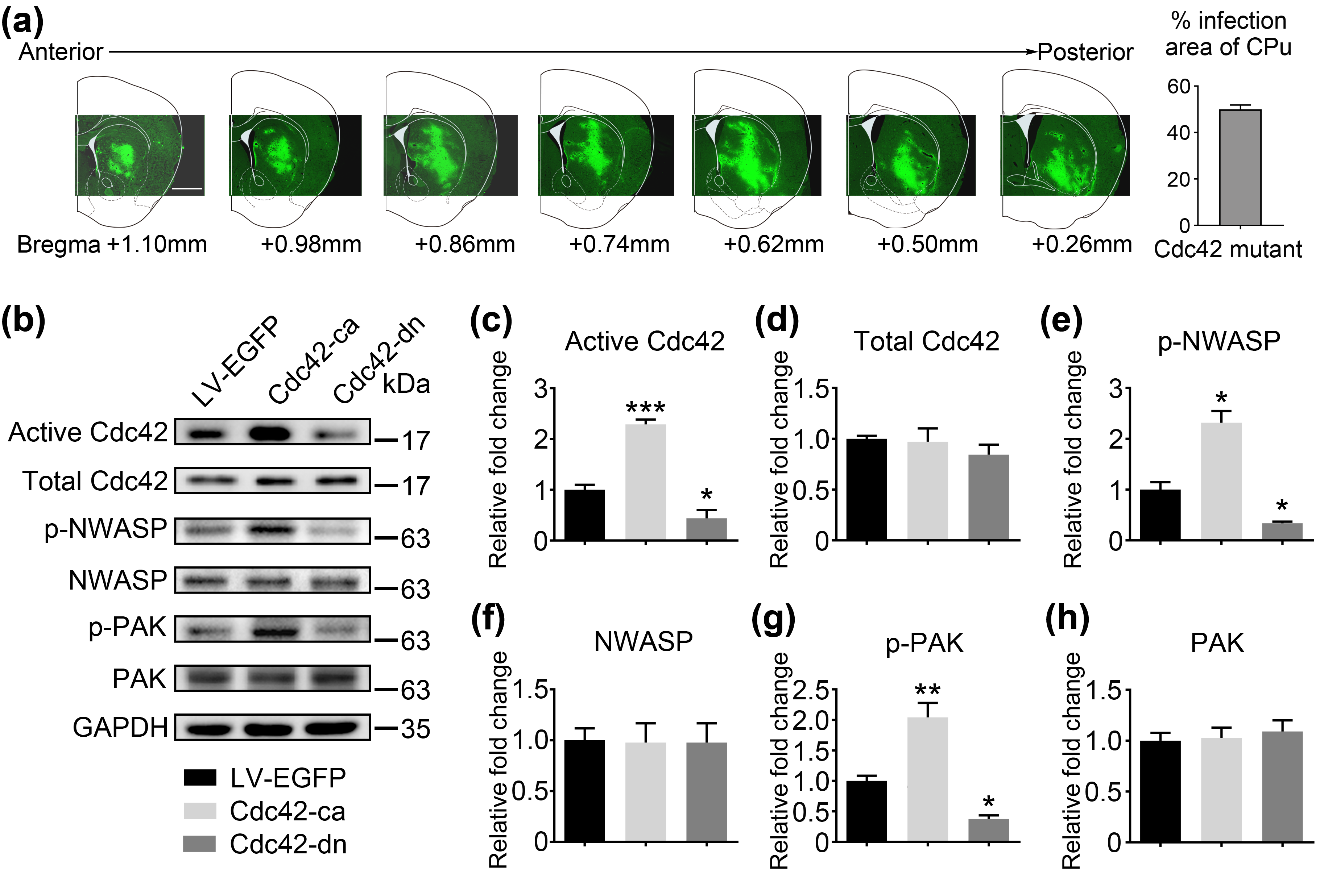


**Figure S2. Cdc42 signaling in the CPu of mice injected with lentiviruses containing Cdc42 mutants**

**(a)** Schematic representation of the infection area in striatal coronal sections (left). Scale bar, 1 mm. The bar represents the percentage of Cdc42 mutant expression in the CPu (right). **(b-h)** Representative western blots (b) and statistical analyses (right) assessing the level of Cdc42 activity and its downstream effectors in the CPu from mice 3 weeks after the injection of lentiviruses. Expression of the Cdc42-ca mutant increased activated Cdc42 (c) and the phosphorylated forms of N-WASP (e), and PAK (g), whereas overexpression of Cdc42-dn decreased activated Cdc42 (c) and the phosphorylated forms of N-WASP (e), and PAK (g) compared to EGFP. Total Cdc42 (d), N-WASP (f), and PAK (h) were unaltered (n = 3-4 mice/group). The data represent the mean ± SEM, and the normal LV-EGFP group was set to 1 for the quantitative analysis, *, p < 0.05, **, p < 0.001, ***, p < 0.0001, one-way ANOVA, followed by Bonferroni correction for multiple comparisons.

**
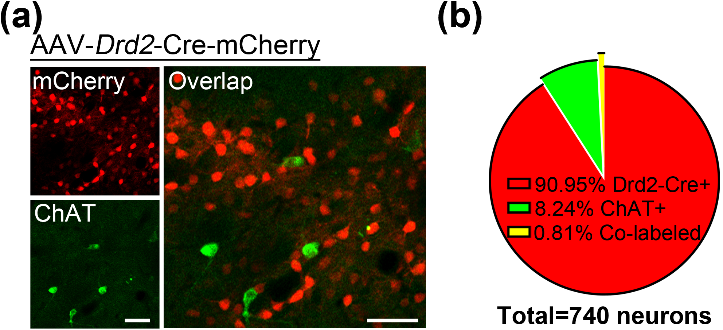
**

**Figure S3. Colocalization of cholinergic interneurons and *Drd2*-Cre-mCherry virus**

**(a)** Representative images showing colocalization of choline acetyltransferase immunoreactivity (green, ChAT) and *Drd2*-Cre-mCherry virus (red) in the CPu. **(b)** Proportion of mCherry, ChAT, and co-labeled cells. Scale bar, 50 μm.


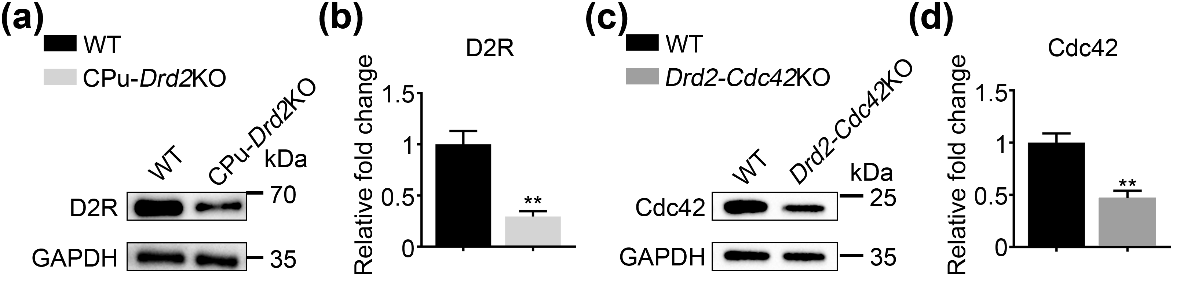


**Figure S4. Verification of the levels of D2R and Cdc42 proteins in the CPu from CPu-*Drd2*KO and *Drd2*-Cdc42KO mice**

**(a and b)** Representative western blots (a) and statistical analyses (b) assessing the level of D2R protein in the CPu from CPu-*Drd2*KO mice. (n = 4 mice/group). The D2R protein in the CPu from CPu-*Drd2*KO mice was decreased (~70%) relative to WT mice. **(c and d)** Representative western blots (c) and statistical analyses (d) assessing the level of Cdc42 protein in the CPu from *Drd2*-Cdc42KO mice. (n = 4 mice/group). Reduced Cdc42 expression (~53%) was observed in the striatum of *Drd2*-Cdc42KO mice compared to WT mice. The data represent the mean ± SEM, and the normal saline group was set to 1 for the quantitative analysis, *, p < 0.05, **, p < 0.001, ***, p < 0.0001 vs. WT mice, two-tailed independent Student's t-tests.

**
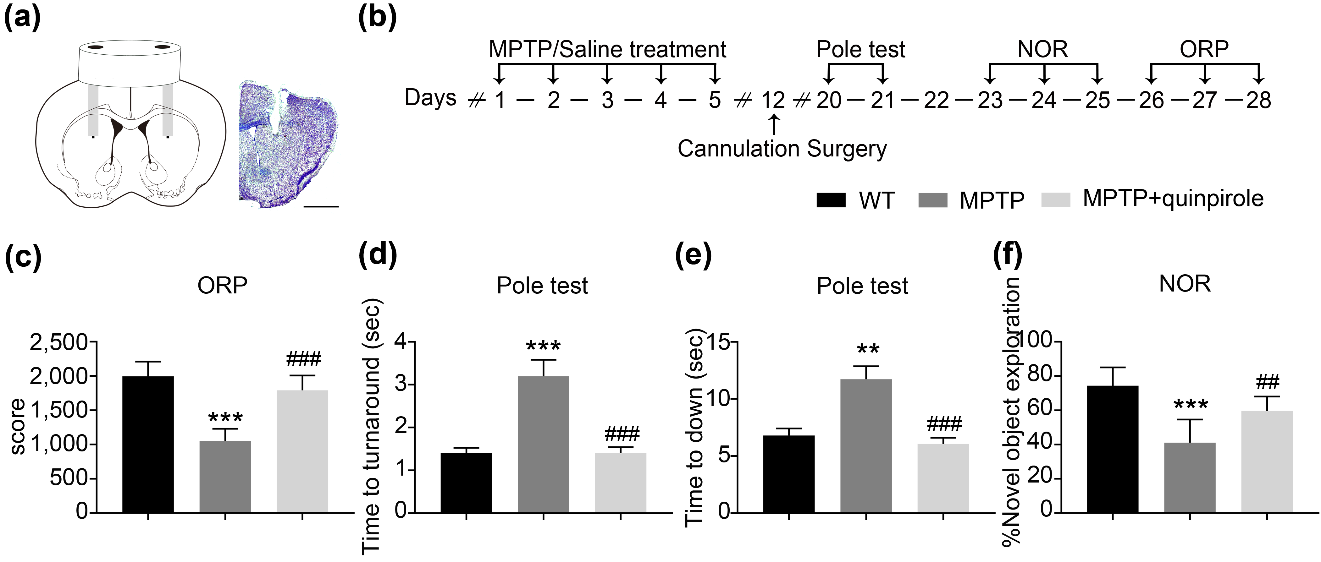
**

**Figure S5. The effects of intra-CPu infusion of quinpirole on the abnormal behavior induced by MPTP treatment**

**(a)** Representative image of the location of the injection cannula tips in the CPu. Scale bar=50 μm. **(b)** Experimental paradigm for cannulation surgery and behavioral process. **(c-f)** Effects of local microinjections of quinpirole into the CPu on behavioral abnormalities induced by MPTP treatment. Motor coordination was assessed by the performance score in ORP (c), and latency to turn around (d) and land on the ground (e) were assessed by the pole test. Cognitive function was measured by the percentage of time spent exploring the novel object in the NOR task (f). All data are expressed as the mean value ± SEM. *, p < 0.05, **, p < 0.001, ***, p < 0.0001, two-way ANOVA, followed by Bonferroni correction for multiple comparisons.


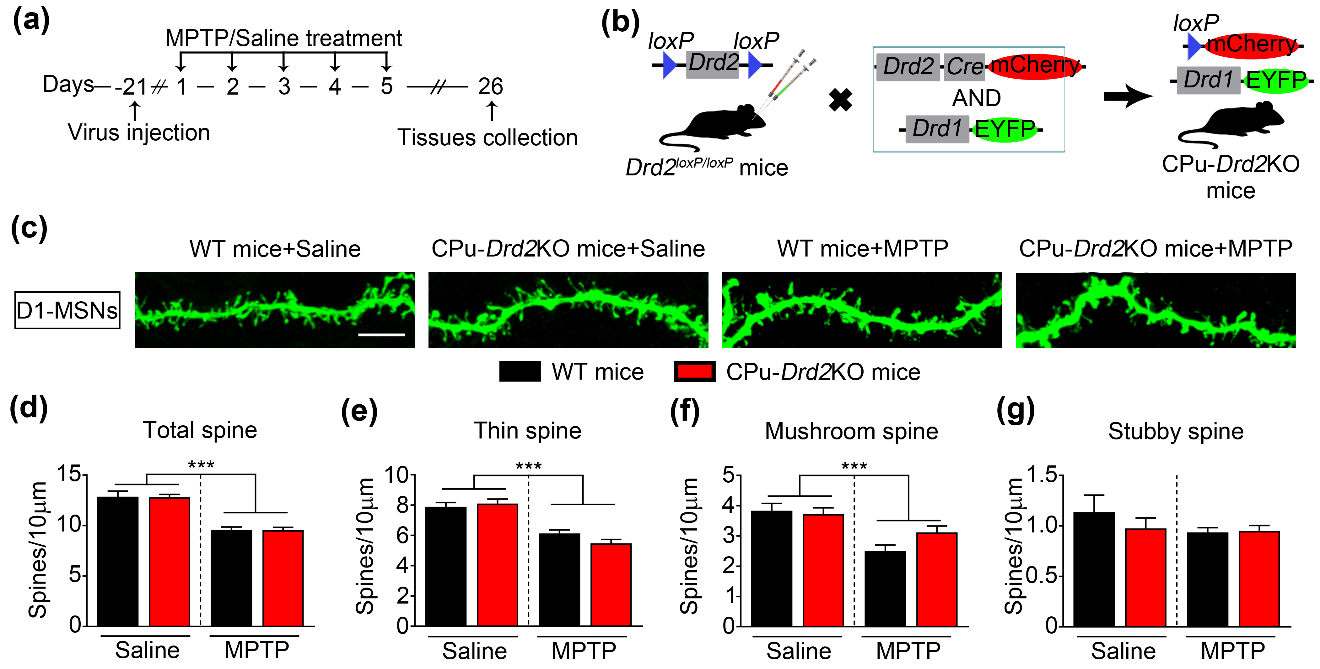


**Figure S6. D1R-MSNs spine density is decreased in CPu-*Drd2*KO mice after MPTP treatment**

**(a)** Timeline for spine analysis for the mice in each group. **(b)** A schematic diagram of the coinjected *Drd2*-Cre recombinase virus and *Drd1*-EYFP in *Drd2^loxp/loxp^* mice. **(c)** Representative image of dendritic spines of D1R-MSNs at 3 weeks. Scale bar, 5 μm. **(d-g)** Statistical analysis of total spine density (d) and the thin (e), mushroom (f), and stubby (g) spine subtypes from virus-transfected D1R-MSNs in the CPu at 3 weeks. The data represent the mean ± SEM. *, p < 0.05, **, p < 0.001, ***, p < 0.0001, two-way ANOVA, followed by Bonferroni correction for multiple comparisons.
