# Supplementary material for: Regulation of Cdc42 signaling by the dopamine D2 receptor in a mouse model of Parkinson’s disease
Source: Aging Cell. 2022 Apr 12;21(5):e13588. doi: 10.1111/acel.13588 (PMC9124300; doi:10.1111/acel.13588)
Supplement: Supplementary file 3 — Table S1‐S17 [file ACEL-21-e13588-s001.docx]

**Table S1. The mouse lines used in this study**

| **Catalog name** | **Catalog #** | **Abbreviation** | **Source** | **Citation** |
| --- | --- | --- | --- | --- |
| B6.129S4(FVB)-*Drd2*tm1.1Mrub/J | JAX020631 | *Drd2^loxp/loxp^* | Purchased from Jax; | (Bello et al., 2011) |
| STOCK *Drd1*tm2.1Stl/J | JAX025700 | *Drd1^loxp/loxp^* | Purchased from Jax; | (Sarinana et al., 2014) |
| *Cdc42*tm1Yizh/J |  | *Cdc42^loxp/loxp^* | Donated by Cincinnati Children's Hospital Medical Center | (Chen et al., 2006) |
| C57BL/6J |  | WT | Obtained from Southern Medical University Animals Center |  |

**Table S2. Genotyping primers and PCR protocol**

| **Gene** | **Forward Primer** | **Reverse Primer** | **Band size (bps)** | **PCR Protocol** |
| --- | --- | --- | --- | --- |
| *Drd1* | TCA TTT CAG GGA ATT GCA G | AAG GGG TCA AAA TTC ACA CA | Mutant=~300  Heterozygote=246 and ~300  Wild type=246 | PCR Protocol 1 _a_ |
| *Drd2* | TCT CCC TCA TCT CTG GAC TCA | TGG GAA AGG GCT ACA GCA | Mutant=~575  Heterozygote=497 and ~575  Wild type=497 | PCR Protocol 2 _b_ |
| *Cdc42* | AGA CAA AAC AAC AAG GTC CAG AAA C | TAC AGT TGG TAC ATA TTC CGA TGG G | WT: 361bp  Loxp/loxp:465bp | PCR Protocol 3 _c_ |

a PCR Protocol 1: 1: 94°C 5 min; 2: 94 °C 20 sec; 3: 60 °C 30 sec; 4: 72 °C 50 sec; 5: Repeat 2-4 for 35 cycles; 6: 72 °C 5 min; 7: 4 °C indefinitely

b PCR Protocol 2: 1: 94 °C 5 min; 2: 94 °C 30 sec; 3: 60 °C 30 sec; 4: 72 °C 1 min; 5: Repeat 2-4 for 40 cycles; 6: 72 °C 5 min; 7: 4 °C indefinitely

c PCR Protocol 3: 1: 94 °C 5 min; 2: 94 °C 30 sec; 3: 60 °C 1 min; 4: 72 °C 1 min; 5: Repeat 2-4 for 33 cycles; 6: 72 °C 8 min; 7: 4 °C indefinitely

**Table S3. Lentivirus and adeno-associated viruses**

| **Viral Vector** | **Promoter** | **Abbreviation** | **Serotype** | **Titer** | **Volume** | **Mouse line** |
| --- | --- | --- | --- | --- | --- | --- |
| Lenti-CMV-EGFP-P2A-MCS-3FLAG-CDC42(N17)_a_ | CMV | Cdc42-dn |  | 3.31E+8 TU/ml | 0.5-0.8ul | WT |
| Lenti-CMV-EGFP-P2A-MCS-3FLAG-CDC42(L61)_a_ | CMV | Cdc42-ca |  | 3.54E+8 TU/ml | 0.5-0.8ul | WT |
| Lenti-CMV-EGFP-P2A-MCS-3FLAG _a_ | CMV | EGFP |  | 4.54E+8 TU/ml | 0.5-0.8ul | WT |
| rAAV-*Drd1*-mCherry-2A-CRE-WPRE-pA _b_ | *Drd1* | *Drd1*-Cre | AAV2/9 | 1.12E+12VG/ml | 0.3-0.5ul | *Drd1^loxp/loxp^* |
| rAAV-*Drd2*-mCherry-2A-CRE-WPRE-pA _b_ | *Drd2* | *Drd2*-Cre | AAV2/9 | 1.29E+12 VG/ml | 0.3-0.5ul | *Drd2^loxp/loxp^* |
| rAAV-*Drd1*-EYFP-WPRE-pA _b_ | *Drd1* | *Drd1-EYFP* | AAV2/9 | 1.01E+12 VG/ml | 0.3-0.5ul | *Drd2^loxp/loxp^* |

a The plasmid used to construct the virus was donated by the Cincinnati Children's Hospital Medical Center and packaged by Obio Technology Corp., Ltd.

b These viruses were obtained from BrainVTA.

**Table S4. Antibody dilutions for immunohistochemistry and immunofluorescence staining**

| **Primary antibodies** | **Host species/dilution** | **Manufacturer/catalog #** |
| --- | --- | --- |
| Anti-GFP | Rabbit, IF, 1:500 | Abcam, ab290 |
| Anti-mCherry | Chicken, IF, 1:1000 | Abcam, ab205402 |
| Anti-D1DR | Mouse, IF, 1:200 | Santa Cruz, sc-33660 |
| Anti-D2 receptor | Rabbit, IF, 1:50 | Millipore, ABN462 |
| Anti-ProDynorphin | Guinea pig, 1:100 | Abcam, ab10280 |
| Anti-met-ENK | Rabbit, IF, 1:100 | Neuromics, RA14124 |
| Anti-ChAT | Goat, IF, 1:100 | Merck Millipore, AB144P |
| **Secondary antibodies** | **Host species/dilution** | **Manufacturer/catalog #** |
| Anti-rabbit IgG | Goat, IHC, drops cover the whole tissue | Boster Biotechnology, SA1022 |
| Anti-rabbit Alexa Fluor 488 | Donkey, IF, 1:200 | Invitrogen, A21206 |
| Anti-mouse Alexa Fluor 488 | Donkey, IF, 1:200 | Invitrogen, A21202 |
| Anti-chicken Alexa Fluor 594 | Donkey, IF, 1:100 | Jackson ImmunoResearch, 703-585-155 |
| Anti-guinea pig Alexa Fluor 488 | Goat, IF, 1:100 | Jackson ImmunoResearch, 106-545-003 |
| Anti-goat Alexa Fluor 594 | Donkey, IF, 1:200 | Invitrogen, A11058 |
| **Staining reagents/kits** | **dilution** | **Manufacturer/catalog #** |
| Hoechst solution | IF, 1 drop/ 1000 μl | Sigma, 94403 |

**Table S5. Antibody dilutions for pull-down assay and western blotting**

| **Reagents** | **dilution** | **Manufacturer/catalog #** |
| --- | --- | --- |
| Rac/Cdc42 Assay Reagent | Pull down assay, 1:100 | Millipore, 14-325 |
| **Primary antibodies** | **Host species/dilution** | **Manufacturer/catalog #** |
| Anti-Cdc42 | Mouse, WB, 1:1000 | BD Transduction Laboratories, 610929 |
| Anti-phospho-PAK1 (Ser199/204)/PAK2 (Ser192/197) | Rabbit, WB, 1:1000 | Cell Signaling Technology, 2605 |
| Anti-PAK1 | Rabbit, WB, 1:1000 | Cell Signaling Technology, 2602 |
| Anti-phospho-Cofilin (Ser3) | Rabbit, WB, 1:1000 | Cell Signaling Technology, 3311 |
| Anti-Cofilin | Rabbit, WB, 1:1000 | Cell Signaling Technology, 3312 |
| Anti-n-wasp (phospho Y256) | Rabbit, WB, 1:10000 | Abcam, ab23395 |
| Anti-n-wasp | Rabbit, WB, 1:1000 | Abcam, ab126626 |
| Anti-GAPDH | Rabbit, WB, 1:3000 | Cwbiotech, CW0101M |
| Anti-D2DR (N-19) | Goat, WB, 1:250 | SantaCruz, sc-7522 |
| **Secondary antibodies** | **Host species/dilution** | **Manufacturer/catalog #** |
| Anti-rabbit IgG, HRP-conjugated | Goat, WB, 1:3000 | Bioss, bs-0295G-HRP |
| Anti-mouse IgG, HRP-conjugated | Goat, WB, 1:3000 | Cwbiotech, CW0102A |
| Anti-goat IgG, HRP-conjugated | Donkey, WB, 1:5000 | Abcam, ab6885 |

**Table S6. Statistical analysis in Figure 1c-j.**

| **Figure** | **Measurement** | **Number of mice** | **Values** | **Statistical Test** |  | **Treatments (p value)** |
| --- | --- | --- | --- | --- | --- | --- |
| Figure 1c | Active Cdc42 | Saline=6 | 1.00±0.26 | One-way ANOVA | F (2, 17) =7.802, p<0.01 ** | Saline vs MPTP 48h, p=0.009 **;  Saline vs MPTP 3w, p=0.014 *;  MPTP 48h vs MPTP 3w, p=1.000 |
|  |  | MPTP 48h=6 | 0.23±0.06 |  |  |  |
|  |  | MPTP 3w=6 | 0.27±0.05 |  |  |  |
| Figure 1d | Total Cdc42 | Saline=6 | 1.00±0.08 |  | F (2, 17) =0.299, p=0.746 |  |
|  |  | MPTP 48h=6 | 0.92±0.07 |  |  |  |
|  |  | MPTP 3w=6 | 0.93±0.08 |  |  |  |
| Figure 1e | Phosphorylation of NWASP | Saline=6 | 1.00±0.12 |  | F (2, 17) =5.502, p<0.05 * | Saline vs MPTP 48h, p=0.040 *;  Saline vs MPTP 3w, p=0.030 *;  MPTP 48h vs MPTP 3w, p=1.000 |
|  |  | MPTP 48h=6 | 0.51±0.07 |  |  |  |
|  |  | MPTP 3w=6 | 0.48±0.16 |  |  |  |
| Figure 1f | NWASP | Saline=6 | 1.00±0.12 |  | F (2, 17) =0.192, p=0.827 |  |
|  |  | MPTP 48h=6 | 1.08±0.10 |  |  |  |
|  |  | MPTP 3w=6 | 1.15±0.26 |  |  |  |
| Figure 1g | Phosphorylation of PAK | Saline=6 | 1.00±0.17 |  | F (2, 17) =14.860, p<0.01 ** | Saline vs MPTP 48h, p<0.001 ***;  Saline vs MPTP 3w, p<0.001 ***;  MPTP 48h vs MPTP 3w, p=1.000 |
|  |  | MPTP 48h=6 | 0.29±0.05 |  |  |  |
|  |  | MPTP 3w=6 | 0.27±0.07 |  |  |  |
| Figure 1h | PAK | Saline=6 | 1.00±0.16 |  | F (2, 17) =0.674, p=0.524 |  |
|  |  | MPTP 48h=6 | 1.15±0.19 |  |  |  |
|  |  | MPTP 3w=6 | 0.90±0.11 |  |  |  |
| Figure 1i | Phosphorylation of Cofilin | Saline=6 | 1.00±0.05 |  | F (2, 17) =9.669, p<0.01 ** | Saline vs MPTP 48h, p=0.003 **;  Saline vs MPTP 3w, p=0.010 *;  MPTP 48h vs MPTP 3w, p=1.000 |
|  |  | MPTP 48h=6 | 0.48±0.10 |  |  |  |
|  |  | MPTP 3w=6 | 0.55±0.11 |  |  |  |
| Figure 1j | Cofilin | Saline=6 | 1.00±0.04 |  | F (2, 17) =0.227, p=0.800 |  |
|  |  | MPTP 48h=6 | 0.94±0.09 |  |  |  |
|  |  | MPTP 3w=6 | 0.93±0.10 |  |  |  |

**Table S7. Statistical analysis in Figure 1o-u.**

| **Figure** | **Measurement** | **Number of spines** | **Values** | **Statistical Test** | **Treatments (p value)** | **simple main effect (p value)** |
| --- | --- | --- | --- | --- | --- | --- |
| Figure 1o | Spine head width | EGFP+Saline=1273 | 0.70±0.00 | Two-way ANOVA | Virus treatment, F (2, 10078) =37.895, p<0.001 ***  Drug treatment, F (2, 10078) =3.042, p=0.048 *  Virus treatment x Drug treatment, F (4, 10078) =5.239, p<0.001 *** | EGFP+Saline vs EGFP+MPTP 48h, p<0.001 *** |
|  |  | Cdc42-ca+Saline=1108 | 0.70±0.00 |  |  | EGFP+Saline vs EGFP+MPTP 3w, p<0.001 *** |
|  |  | Cdc42-dn+Saline=1201 | 0.73±0.00 |  |  | EGFP+Saline vs Cdc42-dn+Saline, p<0.001 *** |
|  |  | EGFP+MPTP 48h=1002 | 0.73±0.00 |  |  | Cdc42-ca+Saline vs Cdc42-dn+Saline, p<0.001 *** |
|  |  | Cdc42-ca+MPTP 48h=1034 | 0.70±0.00 |  |  | EGFP+MPTP 48h vs Cdc42-ca+MPTP 48h, p<0.001 *** |
|  |  | Cdc42-dn+MPTP 48h=1311 | 0.73±0.00 |  |  | Cdc42-ca+MPTP 48h vs Cdc42-dn+MPTP 48h, p<0.001 *** |
|  |  | EGFP+MPTP 3w=929 | 0.73±0.01 |  |  | EGFP+MPTP 3w vs Cdc42-ca+MPTP 3w, p<0.001 *** |
|  |  | Cdc42-ca+MPTP 3w=978 | 0.70±0.00 |  |  | Cdc42-ca+MPTP 3w vs Cdc42-dn+MPTP 3w, p<0.001 *** |
|  |  | Cdc42-dn+MPTP 3w=1242 | 0.73±0.00 |  |  |  |
| Figure 1p | Average spine length | EGFP+Saline=1273 | 1.52±0.01 | Two-way ANOVA | Virus treatment, F (2, 10078) =0.077, p=0.925  Drug treatment, F (2, 10078) =0.478, p=0.620  Virus treatment x Drug treatment, F (4, 10078) =0.093, p=0.985 |  |
|  |  | Cdc42-ca+Saline=1108 | 1.52±0.02 |  |  |  |
|  |  | Cdc42-dn+Saline=1201 | 1.52±0.02 |  |  |  |
|  |  | EGFP+MPTP 48h=1002 | 1.53±0.02 |  |  |  |
|  |  | Cdc42-ca+MPTP 48h=1034 | 1.52±0.02 |  |  |  |
|  |  | Cdc42-dn+MPTP 48h=1311 | 1.52±0.01 |  |  |  |
|  |  | EGFP+MPTP 3w=929 | 1.51±0.02 |  |  |  |
|  |  | Cdc42-ca+MPTP 3w=978 | 1.50±0.02 |  |  |  |
|  |  | Cdc42-dn+MPTP 3w=1242 | 1.52±0.02 |  |  |  |
| Figure 1q | Spine volume | EGFP+Saline=1273 | 0.21±0.00 | Two-way ANOVA | Virus treatment, F (2, 10078) =23.800, p<0.001 ***  Drug treatment, F (2, 10078) =0.637, p=0.529  Virus treatment x Drug treatment, F (4, 10078) =3.575, p<0.01 ** | EGFP+Saline vs EGFP+MPTP 48h, p<0.01 ** |
|  |  | Cdc42-ca+Saline=1108 | 0.21±0.00 |  |  | EGFP+Saline vs EGFP+MPTP 3w, p<0.01 ** |
|  |  | Cdc42-dn+Saline=1201 | 0.24±0.01 |  |  | EGFP+Saline vs Cdc42-dn+Saline, p<0.001 *** |
|  |  | EGFP+MPTP 48h=1002 | 0.23±0.01 |  |  | Cdc42-ca+Saline vs Cdc42-dn+Saline, p<0.001 *** |
|  |  | Cdc42-ca+MPTP 48h=1034 | 0.20±0.01 |  |  | EGFP+MPTP 48h vs Cdc42-ca+MPTP 48h, p<0.001 *** |
|  |  | Cdc42-dn+MPTP 48h=1311 | 0.23±0.00 |  |  | Cdc42-ca+MPTP 48h vs Cdc42-dn+MPTP 48h, p<0.001 *** |
|  |  | EGFP+MPTP 3w=929 | 0.23±0.01 |  |  | EGFP+MPTP 3w vs Cdc42-ca+MPTP 3w, p<0.01 ** |
|  |  | Cdc42-ca+MPTP 3w=978 | 0.20±0.01 |  |  | Cdc42-ca+MPTP 3w vs Cdc42-dn+MPTP 3w, p<0.001 *** |
|  |  | Cdc42-dn+MPTP 3w=1242 | 0.23±0.00 |  |  |  |
| **Figure** | **Measurement** | **Number of dendrites** | **Values** | **Statistical Test** | **Treatments (p value)** | **simple main effect (p value)** |
| Figure 1r | Total spine density | EGFP+Saline=36 | 13.25±0.19 | Two-way ANOVA | Virus treatment, F (2, 206) =29.465, p<0.001 ***  Drug treatment, F (1, 206) =301.541, p<0.001 ***  Virus treatment x Drug treatment, F (2, 206) =5.204, p=0.006 ** | EGFP+Saline vs EGFP+MPTP 48h, p<0.001 *** |
|  |  |  |  |  |  | EGFP+Saline vs EGFP+MPTP 3w, p<0.001 *** |
|  |  | Cdc42-ca+Saline=35 | 13.39±0.23 |  |  | Cdc42-ca+Saline vs Cdc42-ca+MPTP 48h, p<0.001 *** |
|  |  | Cdc42-dn+Saline=35 | 11.57±0.23 |  |  | Cdc42-ca+Saline vs Cdc42-ca+MPTP 3w, p<0.001 *** |
|  |  | EGFP+MPTP 48h=35 | 8.24±0.37 |  |  | Cdc42-dn+Saline vs Cdc42-dn+MPTP 48h, p<0.001 *** |
|  |  | Cdc42-ca+MPTP 48h=30 | 10.22±0.37 |  |  | Cdc42-dn+Saline vs Cdc42-dn+MPTP 3w, p<0.001 *** |
|  |  | Cdc42-dn+MPTP 48h=35 | 7.62±0.30 |  |  | EGFP+Saline vs Cdc42-dn+Saline, p<0.001 *** |
|  |  | EGFP+MPTP 3w=23 | 8.58±0.49 |  |  | Cdc42-ca+Saline vs Cdc42-dn+Saline, p<0.001 *** |
|  |  | Cdc42-ca+MPTP 3w=28 | 10.93±0.37 |  |  | EGFP+MPTP 48h vs Cdc42-ca+MPTP 48h, p<0.001 *** |
|  |  | Cdc42-dn+MPTP 3w=33 | 7.63±0.34 |  |  | Cdc42-ca+MPTP 48h vs Cdc42-dn+MPTP 48h, p<0.001 *** |
|  |  |  |  |  |  | EGFP+MPTP 3w vs Cdc42-ca+MPTP 3w, p<0.001 *** |
|  |  |  |  |  |  | Cdc42-ca+MPTP 3w vs Cdc42-dn+MPTP 3w, p<0.001 *** |
| Figure 1s | Thin spine density | EGFP+Saline=36 | 7.51±0.22 | Two-way ANOVA | Virus treatment, F (2, 290) =31.195, p<0.001 ***  Drug treatment, F (1, 290) =42.560, p<0.001 ***  Virus treatment x Drug treatment, F (2, 290) =2.288, p=0.060 | EGFP+Saline vs EGFP+MPTP 48h, p<0.001 *** |
|  |  | Cdc42-ca+Saline=35 | 7.95±0.34 |  |  | EGFP+Saline vs EGFP+MPTP 3w, p<0.001 *** |
|  |  | Cdc42-dn+Saline=35 | 6.44±0.18 |  |  | Cdc42-dn+Saline vs Cdc42-dn+MPTP 48h, p<0.001 *** |
|  |  | EGFP+MPTP 48h=35 | 4.62±0.23 |  |  | Cdc42-dn+Saline vs Cdc42-dn+MPTP 3w, p<0.001 *** |
|  |  | Cdc42-ca+MPTP 48h=30 | 6.47±0.41 |  |  | EGFP+Saline vs Cdc42-dn+Saline, p<0.05 * |
|  |  | Cdc42-dn+MPTP 48h=35 | 4.57±0.30 |  |  | Cdc42-ca+Saline vs Cdc42-dn+Saline, p<0.01 ** |
|  |  | EGFP+MPTP 3w=23 | 4.85±0.34 |  |  | EGFP+MPTP 48h vs Cdc42-ca+MPTP 48h, p<0.01 ** |
|  |  | Cdc42-ca+MPTP 3w=28 | 6.97±0.41 |  |  | Cdc42-ca+MPTP 48h vs Cdc42-dn+MPTP 48h, p<0.05 * |
|  |  | Cdc42-dn+MPTP 3w=33 | 4.65±0.33 |  |  | EGFP+MPTP 3w vs Cdc42-ca+MPTP 3w, p<0.01 ** |
|  |  |  |  |  |  | Cdc42-ca+MPTP 3w vs Cdc42-dn+MPTP 3w, p<0.01 ** |
| Figure 1t | Mushroom spine density | EGFP+Saline=36 | 4.82±0.24 | Two-way ANOVA | Virus treatment, F (2, 290) =6.025, p=0.003 **  Drug treatment, F (1, 290) =79.993, p<0.001 ***  Virus treatment x Drug treatment, F (2, 290) =0.819, p=0.514 |  |
|  |  | Cdc42-ca+Saline=35 | 4.44±0.29 |  |  |  |
|  |  | Cdc42-dn+Saline=35 | 4.31±0.24 |  |  |  |
|  |  | EGFP+MPTP 48h=35 | 2.70±0.21 |  |  |  |
|  |  | Cdc42-ca+MPTP 48h=30 | 2.82±0.19 |  |  |  |
|  |  | Cdc42-dn+MPTP 48h=35 | 2.21±0.14 |  |  |  |
|  |  | EGFP+MPTP 3w=23 | 2.79±0.25 |  |  |  |
|  |  | Cdc42-ca+MPTP 3w=28 | 2.98±0.22 |  |  |  |
|  |  | Cdc42-dn+MPTP 3w=33 | 2.16±0.13 |  |  |  |
| Figure 1u | Stubby spine density | EGFP+Saline=36 | 0.92±0.06 | Two-way ANOVA | Virus treatment, F (2, 290) =3.335, p=0.037 *  Drug treatment, F (1, 290) =0.065, p=0.937  Virus treatment x Drug treatment, F (2, 290) =0.139, p=0.968 |  |
|  |  | Cdc42-ca+Saline=35 | 1.01±0.06 |  |  |  |
|  |  | Cdc42-dn+Saline=35 | 0.82±0.06 |  |  |  |
|  |  | EGFP+MPTP 48h=35 | 0.92±0.05 |  |  |  |
|  |  | Cdc42-ca+MPTP 48h=30 | 0.93±0.10 |  |  |  |
|  |  | Cdc42-dn+MPTP 48h=35 | 0.84±0.07 |  |  |  |
|  |  | EGFP+MPTP 3w=23 | 0.94±0.06 |  |  |  |
|  |  | Cdc42-ca+MPTP 3w=28 | 0.98±0.11 |  |  |  |
|  |  | Cdc42-dn+MPTP 3w=33 | 0.82±0.06 |  |  |  |

**Table S8. Statistical analysis in Figure 2.**

| **Figure** | **Measurement** | **Number of mice** | **Values** | **Statistical Test** | **Treatments (p value)** | **simple main effect (p value)** |
| --- | --- | --- | --- | --- | --- | --- |
| Figure 2b | Overall Rotarod performance | EGFP+Saline=7 | 2016.57±39.31 | Two-way ANOVA | Virus treatment, F (2, 42) =9.109, p=0.001 ***  Drug treatment, F (1, 42) =33.174, p<0.001 ***  Virus treatment x Drug treatment, F (2, 42) =4.629, p=0.016 * | EGFP+Saline vs EGFP+MPTP, p<0.001 *** |
|  |  | Cdc42-ca+Saline=7 | 2007.86±41.06 |  |  | Cdc42-dn+Saline vs Cdc42-dn+MPTP, p=0.002 ** |
|  |  | Cdc42-dn+Saline=6 | 1758.00±133.11 |  |  | EGFP+MPTP vs Cdc42-ca+MPTP, p<0.001 *** |
|  |  | EGFP+MPTP=7 | 1281.86±121.70 |  |  | Cdc42-ca+MPTP vs Cdc42-dn+MPTP, p=0.001 *** |
|  |  | Cdc42-ca+MPTP=7 | 1849.43±85.38 |  |  |  |
|  |  | Cdc42-dn+MPTP=8 | 1310.50±108.91 |  |  |  |
| Figure 2c | Latency to turn around in the pole test | EGFP+Saline=7 | 1.97±0.21 | Two-way ANOVA | Virus treatment, F (2, 42) =8.912, p=0.001 ***  Drug treatment, F (1, 42) =71.844, p<0.001 ***  Virus treatment x Drug treatment, F (2, 42) =7.146, p=0.002 ** | EGFP+Saline vs EGFP+MPTP, p<0.001 *** |
|  |  |  |  |  |  | Cdc42-ca+Saline vs Cdc42-ca+MPTP, p=0.001 *** |
|  |  | Cdc42-ca+Saline=7 | 2.22±0.20 |  |  | Cdc42-dn+Saline vs Cdc42-dn+MPTP, p=0.005 ** |
|  |  | Cdc42-dn+Saline=6 | 3.08±0.07 |  |  | EGFP+Saline vs Cdc42-dn+Saline, p=0.001 *** |
|  |  | EGFP+MPTP=7 | 4.04±0.18 |  |  | Cdc42-ca+Saline vs Cdc42-dn+Saline, p=0.009 ** |
|  |  | Cdc42-ca+MPTP=7 | 3.18±0.18 |  |  | EGFP+MPTP vs Cdc42-ca+MPTP, p=0.007 ** |
|  |  | Cdc42-dn+MPTP=8 | 3.87±0.20 |  |  | Cdc42-ca+MPTP vs Cdc42-dn+MPTP, p=0.030 * |
| Figure 2d | Latency to land on the ground in the pole test | EGFP+Saline=7 | 5.54±0.29 | Two-way ANOVA | Virus treatment, F (2, 42) =21.410, p<0.001 ***  Drug treatment, F (1, 42) =96.275, p<0.001 ***  Virus treatment x Drug treatment, F (2, 42) =4.811, p=0.014 * | EGFP+Saline vs EGFP+MPTP, p<0.001 *** |
|  |  |  |  |  |  | Cdc42-ca+Saline vs Cdc42-ca+MPTP, p<0.001 *** |
|  |  | Cdc42-ca+Saline=7 | 5.70±0.16 |  |  | Cdc42-dn+Saline vs Cdc42-dn+MPTP, p<0.001 *** |
|  |  | Cdc42-dn+Saline=6 | 7.14±0.38 |  |  | EGFP+Saline vs Cdc42-dn+Saline, p<0.001 *** |
|  |  | EGFP+MPTP=7 | 8.43±0.10 |  |  | Cdc42-ca+Saline vs Cdc42-dn+Saline, p=0.001 *** |
|  |  | Cdc42-ca+MPTP=7 | 7.09±0.11 |  |  | EGFP+MPTP vs Cdc42-ca+MPTP, p=0.002 ** |
|  |  | Cdc42-dn+MPTP=8 | 8.92±0.33 |  |  | Cdc42-ca+MPTP vs Cdc42-dn+MPTP, p<0.001 *** |
| Figure 2e | The percentage of time spent in the novel arm in the Y-maze | EGFP+Saline=7 | 39.86±2.61 | Two-way ANOVA | Virus treatment, F (2, 42) =0.143, p=0.867  Drug treatment, F (1, 42) =12.677, p=0.001 ***  Virus treatment x Drug treatment, F (2, 42) =0.078, p=0.925 |  |
|  |  | Cdc42-ca+Saline=7 | 40.40±2.60 |  |  |  |
|  |  | Cdc42-dn+Saline=6 | 38.76±3.71 |  |  |  |
|  |  | EGFP+MPTP=7 | 32.77±2.25 |  |  |  |
|  |  | Cdc42-ca+MPTP=7 | 31.16±2.61 |  |  |  |
|  |  | Cdc42-dn+MPTP=8 | 30.94±2.80 |  |  |  |
| Figure 2f | The percentage of visits to the novel arm in the Y-maze | EGFP+Saline=7 | 40.99±2.27 | Two-way ANOVA | Virus treatment, F (2, 42) =0.306, p=0.739  Drug treatment, F (1, 42) =25.304, p<0.001 ***  Virus treatment x Drug treatment, F (2, 42) =0.447, p=0.643 |  |
|  |  | Cdc42-ca+Saline=7 | 38.72±1.56 |  |  |  |
|  |  | Cdc42-dn+Saline=6 | 37.51±1.86 |  |  |  |
|  |  | EGFP+MPTP=7 | 30.33±2.41 |  |  |  |
|  |  | Cdc42-ca+MPTP=7 | 30.89±2.11 |  |  |  |
|  |  | Cdc42-dn+MPTP=8 | 30.58±1.94 |  |  |  |
| Figure 2g | The percentage of time exploring the novel object in the NOR task | EGFP+Saline=7 | 71.14±2.97 | Two-way ANOVA | Virus treatment, F (2, 42) =15.213, p<0.001 ***  Drug treatment, F (1, 42) =35.128, p<0.001 ***  Virus treatment x Drug treatment, F (2, 42) =4.469, p=0.018 * | EGFP+Saline vs EGFP+MPTP, p<0.001 *** |
|  |  |  |  |  |  | Cdc42-ca+Saline vs Cdc42-ca+MPTP, p=0.021 * |
|  |  | Cdc42-ca+Saline=7 | 69.48±2.32 |  |  | Cdc42-dn+Saline vs Cdc42-dn+MPTP, p=0.053 |
|  |  | Cdc42-dn+Saline=6 | 57.28±1.66 |  |  | EGFP+Saline vs Cdc42-dn+Saline, p<0.001 *** |
|  |  | EGFP+MPTP=7 | 53.35±2.15 |  |  | Cdc42-ca+Saline vs Cdc42-dn+Saline, p=0.001 *** |
|  |  | Cdc42-ca+MPTP=7 | 62.18±1.42 |  |  | EGFP+MPTP vs Cdc42-ca+MPTP, p=0.018 * |
|  |  | Cdc42-dn+MPTP=8 | 51.15±1.89 |  |  | Cdc42-ca+MPTP vs Cdc42-dn+MPTP, p=0.002 ** |
| Figure 2h | Time spent in open arms in the EPM | EGFP+Saline=7 | 25.12±4.01 | Two-way ANOVA | Virus treatment, F (2, 42) =0.221, p=0.803  Drug treatment, F (1, 42) =0.003, p=0.954  Virus treatment x Drug treatment, F (2, 42) =0.200, p=0.819 |  |
|  |  | Cdc42-ca+Saline=7 | 26.93±4.49 |  |  |  |
|  |  | Cdc42-dn+Saline=6 | 22.19±2.44 |  |  |  |
|  |  | EGFP+MPTP=7 | 24.43±3.67 |  |  |  |
|  |  | Cdc42-ca+MPTP=7 | 24.71±3.84 |  |  |  |
|  |  | Cdc42-dn+MPTP=8 | 24.57±3.04 |  |  |  |
| Figure 2i | Immobility time in the TST | EGFP+Saline=7 | 196.54±13.11 | Two-way ANOVA | Virus treatment, F (2, 42) =0.222, p=0.802  Drug treatment, F (1, 42) =1.881, p=0.179  Virus treatment x Drug treatment, F (2, 42) =0.046, p=0.955 |  |
|  |  | Cdc42-ca+Saline=7 | 197.86±14.17 |  |  |  |
|  |  | Cdc42-dn+Saline=6 | 200.77±11.16 |  |  |  |
|  |  | EGFP+MPTP=7 | 207.57±10.30 |  |  |  |
|  |  | Cdc42-ca+MPTP=7 | 208.65±10.10 |  |  |  |
|  |  | Cdc42-dn+MPTP=8 | 217.79±9.75 |  |  |  |

**Table S9. Statistical analysis in Figure 3.**

| **Figure** | **Measurement** | **Number of mice** | **Values** | **Statistical Test** | **Treatments (p value)** | **simple main effect (p value)** |
| --- | --- | --- | --- | --- | --- | --- |
| Figure 3h | Active Cdc42 | WT+Saline=7 | 1.00±0.02 | Two-way ANOVA | Genotype, F (2, 36) =22.089, p<0.001 ***  Drug treatment, F (1, 36) =15.203, p=0.001 ***  Genotype x Drug treatment, F (2, 36) =7.056, p=0.003 ** | WT+Saline vs WT+MPTP, p<0.001 *** |
|  |  | WT+MPTP=6 | 0.49±0.08 |  |  | CPu-*Drd2*KO+Saline vs CPu-*Drd2*KO+MPTP, p=0.03 * |
|  |  | CPu-*Drd1*KO+Saline=5 | 0.53±0.14 |  |  | WT+Saline vs CPu-*Drd1*KO+Saline, p<0.001 *** |
|  |  | CPu-*Drd1*KO+MPTP=5 | 0.57±0.11 |  |  | WT+Saline vs CPu-*Drd2*KO+Saline, p<0.001 *** |
|  |  | CPu-*Drd2*KO+Saline=6 | 0.40±0.04 |  |  | WT+MPTP vs CPu-*Drd2*KO+MPTP, p=0.01 ** |
|  |  | CPu-*Drd2*KO+MPTP=7 | 0.17±0.03 |  |  | CPu-*Drd1*KO+MPTP vs CPu-*Drd2*KO+MPTP, p=0.002 ** |
| Figure 3j | Active Cdc42 | CPu-*Drd2*KO+Saline=4 | 1.00±0.15 | One-way ANOVA | F (2, 11) =12.552, p=0.002 ** | CPu-*Drd2*KO+Saline vs CPu-*Drd2*KO+MPTP 48h, p=0.030 *;  CPu-*Drd2*KO+Saline vs CPu-*Drd2*KO+MPTP 3w, p=0.002 **;  CPu-*Drd2*KO+MPTP 48h vs CPu-*Drd2*KO+MPTP 3w, p=0.379 |
|  |  | CPu-*Drd2*KO+MPTP 48h=4 | 0.53±0.06 |  |  |  |
|  |  | CPu-*Drd2*KO+MPTP 3w=4 | 0.29±0.07 |  |  |  |
| Figure 3k | Total Cdc42 | CPu-*Drd2*KO+Saline=4 | 1.00±0.08 |  | F (2, 11) =2.837, p=0.111 |  |
|  |  | CPu-*Drd2*KO+MPTP 48h=4 | 1.91±0.07 |  |  |  |
|  |  | CPu-*Drd2*KO+MPTP 3w=4 | 1.23±0.08 |  |  |  |
| Figure 3l | Phosphorylation of NWASP | CPu-*Drd2*KO+Saline=4 | 1.00±0.13 |  | F (2, 11) =8.354, p<0.009 ** | CPu-*Drd2*KO+Saline vs CPu-*Drd2*KO+MPTP 48h, p=0.013 *;  CPu-*Drd2*KO+Saline vs CPu-*Drd2*KO+MPTP 3w, p=0.032 *;  CPu-*Drd2*KO+MPTP 48h vs CPu-*Drd2*KO+MPTP 3w, p=1.000 |
|  |  | CPu-*Drd2*KO+MPTP 48h=4 | 0.56±0.04 |  |  |  |
|  |  | CPu-*Drd2*KO+MPTP 3w=4 | 0.63±0.05 |  |  |  |
| Figure 3m | NWASP | CPu-*Drd2*KO+Saline=4 | 1.00±0.14 |  | F (2, 11) =0.523, p=0.610 |  |
|  |  | CPu-*Drd2*KO+MPTP 48h=4 | 1.14±0.06 |  |  |  |
|  |  | CPu-*Drd2*KO+MPTP 3w=4 | 0.95±0.17 |  |  |  |
| Figure 3n | Phosphorylation of PAK | CPu-*Drd2*KO+Saline=4 | 1.00±0.16 |  | F (2, 11) =10.312, p<0.005 ** | CPu-*Drd2*KO+Saline vs CPu-*Drd2*KO+MPTP 48h, p=0.041 *;  CPu-*Drd2*KO+Saline vs CPu-*Drd2*KO+MPTP 3w, p=0.005 **;  CPu-*Drd2*KO+MPTP 48h vs CPu-*Drd2*KO+MPTP 3w, p=0.608 |
|  |  | CPu-*Drd2*KO+MPTP 48h=4 | 0.46±0.14 |  |  |  |
|  |  | CPu-*Drd2*KO+MPTP 3w=4 | 0.22±0.04 |  |  |  |
| Figure 3o | PAK | CPu-*Drd2*KO+Saline=4 | 1.00±0.17 |  | F (2, 11) =0.873, p=0.450 |  |
|  |  | CPu-*Drd2*KO+MPTP 48h=4 | 1.16±0.14 |  |  |  |
|  |  | CPu-*Drd2*KO+MPTP 3w=4 | 0.28±0.14 |  |  |  |
| Figure 3p | Phosphorylation of Cofilin | CPu-*Drd2*KO+Saline=4 | 1.00±0.08 |  | F (2, 11) =10.118, p<0.005 ** | CPu-*Drd2*KO+Saline vs CPu-*Drd2*KO+MPTP 48h, p=0.006 **;  CPu-*Drd2*KO+Saline vs CPu-*Drd2*KO+MPTP 3w, p=0.023 *;  CPu-*Drd2*KO+MPTP 48h vs CPu-*Drd2*KO+MPTP 3w, p=1.000 |
|  |  | CPu-*Drd2*KO+MPTP 48h=4 | 0.45±0.11 |  |  |  |
|  |  | CPu-*Drd2*KO+MPTP 3w=4 | 0.56±0.08 |  |  |  |
| Figure 3q | Cofilin | CPu-*Drd2*KO+Saline=4 | 1.00±0.12 |  | F (2, 11) =0.215, p=0.811 |  |
|  |  | CPu-*Drd2*KO+MPTP 48h=4 | 0.95±0.05 |  |  |  |
|  |  | CPu-*Drd2*KO+MPTP 3w=4 | 0.92±0.05 |  |  |  |

**Table S10. Statistical analysis in Figure 4.**

| **Figure** | **Measurement** | **Number of spines** | **Values** | **Statistical Test** | **Treatments (p value)** |
| --- | --- | --- | --- | --- | --- |
| Figure 4e | Spine head width | WT+Saline=1384 | 0.68±0.01 | Two-way ANOVA | Genotype, F (1, 7703) =1.293, p=0.255  Drug treatment, F (2, 7703) =30.670, p<0.001 ***  Genotype x Drug treatment, F (1, 7703) =0.575, p=0.563 |
|  |  | CPu-*Drd1*KO+Saline=1313 | 0.67±0.00 |  |  |
|  |  | WT+MPTP 48h=1335 | 0.71±0.00 |  |  |
|  |  | CPu-*Drd1*KO+MPTP 48h=1178 | 0.71±0.00 |  |  |
|  |  | WT+MPTP 3w=1335 | 0.71±0.00 |  |  |
|  |  | CPu-*Drd1*KO+MPTP 3w=1158 | 0.70±0.00 |  |  |
| Figure 4f | Average spine length | WT+Saline=1384 | 1.46±0.02 | Two-way ANOVA | Genotype, F (1, 7703) =1.517, p=0.218  Drug treatment, F (2, 7703) =0.487, p=0.614  Genotype x Drug treatment, F (1, 7703) =0.087, p=0.916 |
|  |  | CPu-*Drd1*KO+Saline=1313 | 1.44±0.01 |  |  |
|  |  | WT+MPTP 48h=1335 | 1.44±0.01 |  |  |
|  |  | CPu-*Drd1*KO+MPTP 48h=1178 | 1.44±0.02 |  |  |
|  |  | WT+MPTP 3w=1335 | 1.44±0.01 |  |  |
|  |  | CPu-*Drd1*KO+MPTP 3w=1158 | 1.44±0.02 |  |  |
| Figure 4g | Spine volume | WT+Saline=1384 | 0.21±0.01 | Two-way ANOVA | Genotype, F (1, 7703) =6.041, p=0.014  Drug treatment, F (2, 7703) =4.387, p=0.012  Genotype x Drug treatment, F (1, 7703) =0.308, p=0.735 |
|  |  | CPu-*Drd1*KO+Saline=1313 | 0.19±0.00 |  |  |
|  |  | WT+MPTP 48h=1335 | 0.22±0.00 |  |  |
|  |  | CPu-*Drd1*KO+MPTP 48h=1178 | 0.21±0.00 |  |  |
|  |  | WT+MPTP 3w=1335 | 0.22±0.00 |  |  |
|  |  | CPu-*Drd1*KO+MPTP 3w=1158 | 0.21±0.00 |  |  |
| **Figure** | **Measurement** | **Number of dendrites** | **Values** | **Statistical Test** | **Treatments (p value)** |
| Figure 4h | Total spine density | WT+Saline=24 | 12.71±0.27 | Two-way ANOVA | Genotype, F (1, 130) =2.875 p=0.092  Drug treatment, F (2, 130) =41.208, p<0.001 ***  Genotype x Drug treatment, F (2, 130) =0.890, p=0.413 |
|  |  | CPu-*Drd1*KO+Saline=22 | 12.01±0.24 |  |  |
|  |  | WT+MPTP 48h=22 | 10.62±0.25 |  |  |
|  |  | CPu-*Drd1*KO+MPTP 48h=22 | 10.53±0.28 |  |  |
|  |  | WT+MPTP 3w=20 | 10.45±0.22 |  |  |
|  |  | CPu-*Drd1*KO+MPTP 3w=20 | 10.23±0.18 |  |  |
| Figure 4i | Thin spine density | WT+Saline=24 | 8.67±0.20 | Two-way ANOVA | Genotype, F (1, 130) =2.007 p=0.159  Drug treatment, F (2, 130) =20.351, p<0.001 ***  Genotype x Drug treatment, F (2, 130) =0.142, p=0.868 |
|  |  | CPu-*Drd1*KO+Saline=22 | 8.46±0.15 |  |  |
|  |  | WT+MPTP 48h=22 | 7.66±0.21 |  |  |
|  |  | CPu-*Drd1*KO+MPTP 48h=22 | 7.53±0.22 |  |  |
|  |  | WT+MPTP 3w=20 | 7.61±0.21 |  |  |
|  |  | CPu-*Drd1*KO+MPTP 3w=20 | 7.28±0.14 |  |  |
| Figure 4j | Mushroom spine density | WT+Saline=24 | 3.11±0.18 | Two-way ANOVA | Genotype, F (1, 130) =0.756 p=0.386  Drug treatment, F (2, 130) =22.743, p<0.001 ***  Genotype x Drug treatment, F (2, 130) =2.910, p=0.058 |
|  |  | CPu-*Drd1*KO+Saline=22 | 2.61±0.16 |  |  |
|  |  | WT+MPTP 48h=22 | 2.03±0.16 |  |  |
|  |  | CPu-*Drd1*KO+MPTP 48h=22 | 2.07±0.10 |  |  |
|  |  | WT+MPTP 3w=20 | 1.90±0.14 |  |  |
|  |  | CPu-*Drd1*KO+MPTP 3w=20 | 2.05±0.11 |  |  |
| Figure 4k | Stubby spine density | WT+Saline=24 | 0.94±0.06 | Two-way ANOVA | Genotype, F (1, 130) =0.110 p=0.741  Drug treatment, F (2, 130) =0.064, p=0.938  Genotype x Drug treatment, F (2, 130) =0.076, p=0.927 |
|  |  | CPu-*Drd1*KO+Saline=22 | 0.94±0.05 |  |  |
|  |  | WT+MPTP 48h=22 | 0.94±0.04 |  |  |
|  |  | CPu-*Drd1*KO+MPTP 48h=22 | 0.93±0.04 |  |  |
|  |  | WT+MPTP 3w=20 | 0.94±0.05 |  |  |
|  |  | CPu-*Drd1*KO+MPTP 3w=20 | 0.90±0.04 |  |  |

**Table S11. Statistical analysis in Figure 5.**

| **Figure** | **Measurement** | **Number of spines** | **Values** | **Statistical Test** | **Treatments (p value)** | **simple main effect (p value)** |
| --- | --- | --- | --- | --- | --- | --- |
| Figure 5f | Spine head width | WT+Saline=1161 | 0.68±0.00 | Two-way ANOVA | Genotype, F (2, 9444) =30.383, p<0.001 ***  Drug treatment, F (2, 9444) =6.983, p<0.001 ***  Genotype x Drug treatment, F (4, 9444) =4.097, p=0.003 ** | WT+Saline vs WT+MPTP 48h, p<0.001 *** |
|  |  | CPu-*Drd2*KO+Saline=953 | 0.71±0.01 |  |  | WT+Saline vs WT+MPTP 3w, p<0.001 *** |
|  |  | CPu-*Drd2*KO+Cdc42-ca+Saline=1201 | 0.68±0.01 |  |  | WT+Saline vs CPu-*Drd2*KO+Saline, p<0.001 *** |
|  |  | WT+MPTP 48h=947 | 0.72±0.01 |  |  | CPu-*Drd2*KO+Saline vs CPu-*Drd2*KO+Cdc42-ca+Saline, p<0.001 *** |
|  |  | CPu-*Drd2*KO+MPTP 48h=1069 | 0.72±0.01 |  |  | WT+MPTP 48h vs CPu-*Drd2*KO+Cdc42-ca+MPTP 48h, p<0.001 *** |
|  |  | CPu-*Drd2*KO+Cdc42-ca+MPTP 48h=1097 | 0.68±0.01 |  |  | CPu-*Drd2*KO+MPTP 48h vs CPu-*Drd2*KO+Cdc42-ca+MPTP 48h, p<0.001 *** |
|  |  | WT+MPTP 3w=925 | 0.72±0.01 |  |  | WT+MPTP 3w vs CPu-*Drd2*KO+Cdc42-ca+MPTP 3w, p<0.001 *** |
|  |  | CPu-*Drd2*KO+MPTP 3w=1035 | 0.72±0.01 |  |  | CPu-*Drd2*KO+MPTP 3w vs CPu-*Drd2*KO+Cdc42-ca+MPTP 3w, p<0.001 *** |
|  |  | CPu-*Drd2*KO+Cdc42-ca+MPTP 3w=1056 | 0.68±0.01 |  |  |  |
| Figure 5g | Average spine length | WT+Saline=1161 | 1.45±0.02 | Two-way ANOVA | Genotype, F (2, 9444) =2.244, p=0.106  Drug treatment, F (2, 9444) =0.159, p=0.853  Genotype x Drug treatment, F (4, 9444) =0.330, p=0.858 |  |
|  |  | CPu-*Drd2*KO+Saline=953 | 1.44±0.02 |  |  |  |
|  |  | CPu-*Drd2*KO+Cdc42-ca+Saline=1201 | 1.49±0.02 |  |  |  |
|  |  | WT+MPTP 48h=947 | 1.45±0.02 |  |  |  |
|  |  | CPu-*Drd2*KO+MPTP 48h=1069 | 1.45±0.02 |  |  |  |
|  |  | CPu-*Drd2*KO+Cdc42-ca+MPTP 48h=1097 | 1.48±0.02 |  |  |  |
|  |  | WT+MPTP 3w=925 | 1.45±0.02 |  |  |  |
|  |  | CPu-*Drd2*KO+MPTP 3w=1035 | 1.47±0.02 |  |  |  |
|  |  | CPu-*Drd2*KO+Cdc42-ca+MPTP 3w=1056 | 1.48±0.02 |  |  |  |
| Figure 5h | Spine volume | WT+Saline=1161 | 0.20±0.00 | Two-way ANOVA | Genotype, F (2, 9444) =31.611, p<0.001 ***  Drug treatment, F (2, 9444) =5.845, p=0.003 **  Genotype x Drug treatment, F (4, 9444) =5.108, p<0.001 *** | WT+Saline vs WT+MPTP 48h, p<0.001 *** |
|  |  | CPu-*Drd2*KO+Saline=953 | 0.24±0.01 |  |  | WT+Saline vs WT+MPTP 3w, p<0.001 *** |
|  |  | CPu-*Drd2*KO+Cdc42-ca+Saline=1201 | 0.21±0.01 |  |  | WT+Saline vs CPu-*Drd2*KO+Saline, p<0.001 *** |
|  |  | WT+MPTP 48h=947 | 0.24±0.01 |  |  | CPu-*Drd2*KO+Saline vs CPu-*Drd2*KO+Cdc42-ca+Saline, p<0.001 *** |
|  |  | CPu-*Drd2*KO+MPTP 48h=1069 | 0.24±0.01 |  |  | WT+MPTP 48h vs CPu-*Drd2*KO+Cdc42-ca+MPTP 48h, p<0.001 *** |
|  |  | CPu-*Drd2*KO+Cdc42-ca+MPTP 48h=1097 | 0.20±0.00 |  |  | CPu-*Drd2*KO+MPTP 48h vs CPu-*Drd2*KO+Cdc42-ca+MPTP 48h, p<0.001 *** |
|  |  | WT+MPTP 3w=925 | 0.24±0.01 |  |  | WT+MPTP 3w vs CPu-*Drd2*KO+Cdc42-ca+MPTP 3w, p<0.001 *** |
|  |  | CPu-*Drd2*KO+MPTP 3w=1035 | 0.25±0.01 |  |  | CPu-*Drd2*KO+MPTP 3w vs CPu-*Drd2*KO+Cdc42-ca+MPTP 3w, p<0.001 *** |
|  |  | CPu-*Drd2*KO+Cdc42-ca+MPTP 3w=1056 | 0.20±0.00 |  |  |  |
| **Figure** | **Measurement** | **Number of dendrites** | **Values** | **Statistical Test** | **Treatments (p value)** | **simple main effect (p value)** |
| Figure 5i | Total spine density | WT+Saline=25 | 12.28±0.15 | Two-way ANOVA | Genotype, F (2, 211) =24.688, p<0.001 ***  Drug treatment, F (2, 211) =139.887, p<0.001 ***  Genotype x Drug treatment, F (4, 211) =18.349, p<0.001 *** | WT+Saline vs WT+MPTP 48h, p<0.001 *** |
|  |  | CPu-*Drd2*KO+Saline=25 | 9.05±0.14 |  |  | WT+Saline vs WT+MPTP 3w, p<0.001 *** |
|  |  | CPu-*Drd2*KO+Cdc42-ca+Saline=29 | 10.53±0.1910.53±0.19 |  |  | CPu-*Drd2*KO+Saline vs CPu-*Drd2*KO+MPTP 48h, p<0.001 *** |
|  |  |  |  |  |  | CPu-*Drd2*KO+Saline vs CPu-*Drd2*KO+MPTP 3w, p<0.001 *** |
|  |  | WT+MPTP 48h=27 | 7.51±0.20 |  |  | CPu-*Drd2*KO+Cdc42-ca+Saline vs CPu-*Drd2*KO+Cdc42-ca+MPTP 48h, p<0.001 *** |
|  |  |  |  |  |  | CPu-*Drd2*KO+Cdc42-ca+Saline vs CPu-*Drd2*KO+Cdc42-ca+MPTP 3w, p<0.001 *** |
|  |  | CPu-*Drd2*KO+MPTP 48h=21 | 7.36±0.28 |  |  | WT+Saline vs CPu-*Drd2*KO+Saline, p<0.001 *** |
|  |  |  |  |  |  | WT+Saline vs CPu-*Drd2*KO+Cdc42-ca+Saline, p<0.001 *** |
|  |  | CPu-*Drd2*KO+Cdc42-ca+MPTP 48h=21 | 8.63±0.36 |  |  | CPu-*Drd2*KO+Saline vs CPu-*Drd2*KO+Cdc42-ca+Saline, p<0.001 *** |
|  |  |  |  |  |  | WT++MPTP 48h vs CPu-*Drd2*KO+Cdc42-ca++MPTP 48h, p<0.001 *** |
|  |  | WT+MPTP 3w=27 | 7.60±0.20 |  |  | CPu-*Drd2*KO++MPTP 48h vs CPu-*Drd2*KO+Cdc42-ca++MPTP 48h, p<0.001 *** |
|  |  | CPu-*Drd2*KO+MPTP 3w=21 | 7.58±0.25 |  |  | WT++MPTP 3w vs CPu-*Drd2*KO+Cdc42-ca++MPTP 3w, p<0.001 *** |
|  |  | CPu-*Drd2*KO+Cdc42-ca+MPTP 3w=21 | 8.58±0.36 |  |  | CPu-*Drd2*KO++MPTP 3w vs CPu-*Drd2*KO+Cdc42-ca++MPTP 3w, p<0.001 *** |
| Figure 5j | Thin spine density | WT+Saline=25 | 7.55±0.11 | Two-way ANOVA | Genotype, F (2, 211) =30.877, p<0.001 ***  Drug treatment, F (2, 211) =54.914, p<0.001 ***  Genotype x Drug treatment, F (4, 211) =17.691, p<0.001 *** | WT+Saline vs WT+MPTP 48h, p<0.001 *** |
|  |  |  |  |  |  | WT+Saline vs WT+MPTP 3w, p<0.001 *** |
|  |  | CPu-*Drd2*KO+Saline=25 | 4.65±0.19 |  |  | CPu-*Drd2*KO+Cdc42-ca+Saline vs CPu-*Drd2*KO+Cdc42-ca+MPTP 48h, p<0.05 * |
|  |  | CPu-*Drd2*KO+Cdc42-ca+Saline=29 | 6.27±0.17 |  |  | CPu-*Drd2*KO+Cdc42-ca+Saline vs CPu-*Drd2*KO+Cdc42-ca+MPTP 3w, p<0.05 * |
|  |  | WT+MPTP 48h=27 | 3.96±0.14 |  |  | WT+Saline vs CPu-*Drd2*KO+Saline, p<0.001 *** |
|  |  | CPu-*Drd2*KO+MPTP 48h=21 | 3.99±0.27 |  |  | WT+Saline vs CPu-*Drd2*KO+Cdc42-ca+Saline, p<0.001 *** |
|  |  | CPu-*Drd2*KO+Cdc42-ca+MPTP 48h=21 | 5.48±0.38 |  |  | CPu-*Drd2*KO+Saline vs CPu-*Drd2*KO+Cdc42-ca+Saline, p<0.001 *** |
|  |  | WT+MPTP 3w=27 | 4.11±0.15 |  |  | WT+MPTP 48h vs CPu-*Drd2*KO+Cdc42-ca+MPTP 48h, p<0.001 *** |
|  |  |  |  |  |  | CPu-*Drd2*KO+MPTP 48h vs CPu-*Drd2*KO+Cdc42-ca+MPTP 48h, p<0.001 *** |
|  |  | CPu-*Drd2*KO+MPTP 3w=21 | 4.12±0.22 |  |  | WT+MPTP 3w vs CPu-*Drd2*KO+Cdc42-ca+MPTP 3w, p<0.001 *** |
|  |  | CPu-*Drd2*KO+Cdc42-ca+MPTP 3w=21 | 5.38±0.37 |  |  | CPu-*Drd2*KO+MPTP 3w vs CPu-*Drd2*KO+Cdc42-ca+MPTP 3w, p<0.001 *** |
| Figure 5k | Mushroom spine density | WT+Saline=25 | 3.88±0.15 | Two-way ANOVA | Genotype, F (2, 211) =5.866, p<0.003 **  Drug treatment, F (2, 211) =68.883, p<0.001 ***  Genotype x Drug treatment, F (4, 211) =0.176, p=0.950 |  |
|  |  | CPu-*Drd2*KO+Saline=25 | 3.75±0.13 |  |  |  |
|  |  | CPu-*Drd2*KO+Cdc42-ca+Saline=29 | 3.49±0.15 |  |  |  |
|  |  | WT+MPTP 48h=27 | 2.68±0.14 |  |  |  |
|  |  | CPu-*Drd2*KO+MPTP 48h=21 | 2.55±0.16 |  |  |  |
|  |  | CPu-*Drd2*KO+Cdc42-ca+MPTP 48h=21 | 2.26±0.15 |  |  |  |
|  |  | WT+MPTP 3w=27 | 2.55±0.15 |  |  |  |
|  |  | CPu-*Drd2*KO+MPTP 3w=21 | 2.62±0.18 |  |  |  |
|  |  | CPu-*Drd2*KO+Cdc42-ca+MPTP 3w=21 | 2.17±0.15 |  |  |  |
| Figure 5l | Stubby spine density | WT+Saline=25 | 0.93±0.06 | Two-way ANOVA | Genotype, F (2, 211) =1.883, p=1.155  Drug treatment, F (2, 211) =0.925, p=0.397  Genotype x Drug treatment, F (4, 211) =0.443, p=0.777 |  |
|  |  | CPu-*Drd2*KO+Saline=25 | 0.82±0.06 |  |  |  |
|  |  | CPu-*Drd2*KO+Cdc42-ca+Saline=29 | 0.85±0.05 |  |  |  |
|  |  | WT+MPTP 48h=27 | 0.87±0.07 |  |  |  |
|  |  | CPu-*Drd2*KO+MPTP 48h=21 | 0.83±0.05 |  |  |  |
|  |  | CPu-*Drd2*KO+Cdc42-ca+MPTP 48h=21 | 0.90±0.04 |  |  |  |
|  |  | WT+MPTP 3w=27 | 0.95±0.09 |  |  |  |
|  |  | CPu-*Drd2*KO+MPTP 3w=21 | 0.84±0.06 |  |  |  |
|  |  | CPu-*Drd2*KO+Cdc42-ca+MPTP 3w=21 | 0.99±0.05 |  |  |  |
| **Figure** | **Measurement** | **Number of mice** | **Values** | **Statistical Test** | **Treatments (p value)** | **simple main effect (p value)** |
| Figure 5m | Overall Rotarod performance | WT+Saline=8 | 1978.75±47.76 | Two-way ANOVA | Genotype, F (2, 45) =12.889, p<0.001 ***  Drug treatment, F (1, 45) =24.665, p<0.001 ***  Genotype x Drug treatment, F (2, 45) =4.905, p=0.013 * | WT+Saline vs WT+MPTP, p<0.001 *** |
|  |  | CPu-*Drd2*KO+Saline=8 | 1679.50±88.20 |  |  | CPu-*Drd2*KO+Saline vs CPu-*Drd2*KO+MPTP, p=0.010 * |
|  |  | CPu-*Drd2*KO+Cdc42-ca+Saline=9 | 1975.22±44.24 |  |  | WT+Saline vs CPu-*Drd2*KO+Saline, p=0.041 * |
|  |  | WT+MPTP=6 | 1362.33±157.04 |  |  | CPu-*Drd2*KO+Saline vs CPu-*Drd2*KO+Cdc42-ca+Saline, p=0.037 * |
|  |  | CPu-*Drd2*KO+MPTP=6 | 1342.00±127.85 |  |  | WT+MPTP vs CPu-*Drd2*KO+Cdc42-ca+MPTP, p<0.001 *** |
|  |  | CPu-*Drd2*KO+Cdc42-ca+MPTP=8 | 1886.38±54.04 |  |  | CPu-*Drd2*KO+MPTP vs CPu-*Drd2*KO+Cdc42-ca+MPTP , p<0.001 *** |
| Figure 5n | Latency to turn around in the pole test | WT+Saline=8 | 3.38±0.18 | Two-way ANOVA | Genotype, F (2, 44) =8.186, p=0.001 ***  Drug treatment, F (1, 44) =8.685, p=0.005 **  Genotype x Drug treatment, F (2, 44) =6.294, p<0.004 ** | WT+Saline vs WT+MPTP, p<0.001 *** |
|  |  | CPu-*Drd2*KO+Saline=8 | 4.82±0.22 |  |  | WT+Saline vs CPu-*Drd2*KO+Saline, p=0.004 ** |
|  |  | CPu-*Drd2*KO+Cdc42-ca+Saline=7 | 3.55±0.35 |  |  | CPu-*Drd2*KO+Saline vs CPu-*Drd2*KO+Cdc42-ca+Saline, p=0.018 * |
|  |  | WT+MPTP=8 | 5.39±0.23 |  |  | WT+MPTP vs CPu-*Drd2*KO+Cdc42-ca+MPTP, p=0.002 ** |
|  |  | CPu-*Drd2*KO+MPTP=7 | 4.97±0.53 |  |  | CPu-*Drd2*KO+MPTP vs CPu-*Drd2*KO+Cdc42-ca+MPTP , p=0.024 * |
|  |  | CPu-*Drd2*KO+Cdc42-ca+MPTP=6 | 3.66±0.29 |  |  |  |
| Figure 5o | Latency to land on the ground in the pole test | WT+Saline=8 | 5.97±0.32 | Two-way ANOVA | Genotype, F (2, 44) =29.093, p=0.001 ***  Drug treatment, F (1, 44) =37.202, p<0.001 ***  Genotype x Drug treatment, F (2, 44) =14.616, p<0.001 *** | WT+Saline vs WT+MPTP, p<0.001 *** |
|  |  | CPu-*Drd2*KO+Saline=8 | 11.66±0.77 |  |  | WT+Saline vs CPu-*Drd2*KO+Saline, p<0.001 *** |
|  |  | CPu-*Drd2*KO+Cdc42-ca+Saline=7 | 7.57±0.37 |  |  | CPu-*Drd2*KO+Saline vs CPu-*Drd2*KO+Cdc42-ca+Saline, p<0.001 *** |
|  |  | WT+MPTP=8 | 11.62±0.42 |  |  | WT+MPTP vs CPu-*Drd2*KO+Cdc42-ca+MPTP, p=0.005 ** |
|  |  | CPu-*Drd2*KO+MPTP=7 | 12.22±0.52 |  |  | CPu-*Drd2*KO+MPTP vs CPu-*Drd2*KO+Cdc42-ca+MPTP , p=0.001 *** |
|  |  | CPu-*Drd2*KO+Cdc42-ca+MPTP=6 | 9.08±0.51 |  |  |  |
| Figure 5p | The percentage of time exploring the novel object in the NOR task | WT+Saline=9 | 82.22±1.98 | Two-way ANOVA | Genotype, F (2, 42) =19.932, p<0.001 ***  Drug treatment, F (1, 42) =56.952, p<0.001 ***  Genotype x Drug treatment, F (2, 42) =14.884, p<0.001 *** | WT+Saline vs WT+MPTP, p<0.001 *** |
|  |  |  |  |  |  | CPu-*Drd2*KO+Saline vs CPu-*Drd2*KO+MPTP, p=0.052 |
|  |  |  |  |  |  | CPu-*Drd2*KO+Cdc42-ca+Saline vs CPu-*Drd2*KO+Cdc42-ca+MPTP, p=0.029 * |
|  |  | CPu-*Drd2*KO+Saline=9 | 60.26±2.36 |  |  | WT+Saline vs CPu-*Drd2*KO+Saline, p<0.001 *** |
|  |  | CPu-*Drd2*KO+Cdc42-ca+Saline=6 | 74.04±1.30 |  |  | CPu-*Drd2*KO+Saline vs CPu-*Drd2*KO+Cdc42-ca+Saline, p<0.001 *** |
|  |  | WT+MPTP=6 | 54.42±1.57 |  |  | WT+Saline vs CPu-*Drd2*KO+Cdc42-ca+Saline, p=0.036 * |
|  |  | CPu-*Drd2*KO+MPTP=6 | 54.05±2.85 |  |  | WT+MPTP vs CPu-*Drd2*KO+Cdc42-ca+MPTP, p=0.004 ** |
|  |  | CPu-*Drd2*KO+Cdc42-ca+MPTP=6 | 66.35±2.49 |  |  | CPu-*Drd2*KO+MPTP vs CPu-*Drd2*KO+Cdc42-ca+MPTP , p=0.003 ** |

**Table S12. Statistical analysis in Figure 6.**

| **Figure** | **Measurement** | **Number of spines** | **Values** | **Statistical Test** | **Treatments (p value)** | **simple main effect (p value)** |
| --- | --- | --- | --- | --- | --- | --- |
| Figure 6d | Spine head width | WT+Saline=1222 | 0.70±0.00 | Two-way ANOVA | Genotype, F (1, 5897) =1.367, p=0.242  Drug treatment, F (2, 5897) =6.847, p=0.001 ***  Genotype x Drug treatment, F (2, 5897) =5.555, p=0.004 ** | WT+Saline vs WT+MPTP 48h, p<0.01 *** |
|  |  | *Drd2*-Cdc42KO+Saline=928 | 0.72±0.01 |  |  | WT+Saline vs WT+MPTP 3w, p<0.01 *** |
|  |  | WT+MPTP 48h=807 | 0.73±0.01 |  |  | WT+Saline vs *Drd2*-Cdc42KO+Saline, p<0.01 *** |
|  |  | *Drd2*-Cdc42KO+MPTP 48h=1089 | 0.72±0.00 |  |  |  |
|  |  | WT+MPTP 3w=815 | 0.73±0.01 |  |  |  |
|  |  | *Drd2*-Cdc42KO+MPTP 3w=1036 | 0.73±0.00 |  |  |  |
| Figure 6e | Average spine length | WT+Saline=1222 | 1.51±0.02 | Two-way ANOVA | Genotype, F (1, 5897) =0.453, p=0.501  Drug treatment, F (2, 5897) =0.114, p=0.892  Genotype x Drug treatment, F (2, 5897) =1.197, p=0.302 |  |
|  |  | *Drd2*-Cdc42KO+Saline=928 | 1.49±0.02 |  |  |  |
|  |  | WT+MPTP 48h=807 | 1.49±0.02 |  |  |  |
|  |  | *Drd2*-Cdc42KO+MPTP 48h=1089 | 1.49±0.02 |  |  |  |
|  |  | WT+MPTP 3w=815 | 1.48±0.02 |  |  |  |
|  |  | *Drd2*-Cdc42KO+MPTP 3w=1036 | 1.52±0.02 |  |  |  |
| Figure 6f | Spine volume | WT+Saline=1222 | 0.22±0.01 | Two-way ANOVA | Genotype, F (1, 5897) =0.011, p=0.915  Drug treatment, F (2, 5897) =1.903, p=0.149 *  Genotype x Drug treatment, F (2, 5897) =4.600, p=0.010 ** | WT+Saline vs WT+MPTP 48h, p<0.01 ** |
|  |  | *Drd2*-Cdc42KO+Saline=928 | 0.24±0.01 |  |  | WT+Saline vs WT+MPTP 3w, p<0.05 * |
|  |  | WT+MPTP 48h=807 | 0.24±0.01 |  |  | WT+Saline vs *Drd2*-Cdc42KO+Saline, p<0.05 * |
|  |  | *Drd2*-Cdc42KO+MPTP 48h=1089 | 0.23±0.01 |  |  |  |
|  |  | WT+MPTP 3w=815 | 0.24±0.01 |  |  |  |
|  |  | *Drd2*-Cdc42KO+MPTP 3w=1036 | 0.23±0.01 |  |  |  |
| **Figure** | **Measurement** | **Number of dendrites** | **Values** | **Statistical Test** | **Treatments (p value)** | **simple main effect (p value)** |
| Figure 6g | Total spine density | WT+Saline=25 | 12.32±0.15 | Two-way ANOVA | Genotype, F (1, 144) =45.118, p<0.001 ***  Drug treatment, F (1, 144) =105.623, p<0.001 ***  Genotype x Drug treatment, F (1, 144) =29.114, p<0.001 *** | WT+Saline vs WT+MPTP 48h, p<0.01 *** |
|  |  | *Drd2*-Cdc42KO+Saline=27 | 8.72±0.19 |  |  | WT+Saline vs WT+MPTP 3w, p<0.01 *** |
|  |  | WT+MPTP=27 | 7.51±0.20 |  |  | *Drd2*-Cdc42KO+Saline vs *Drd2*-Cdc42KO+MPTP 48h, p<0.01 *** |
|  |  | *Drd2*-Cdc42KO+MPTP=24 | 7.12±0.36 |  |  | *Drd2*-Cdc42KO+Saline vs *Drd2*-Cdc42KO+MPTP 3w, p<0.01 *** |
|  |  | WT+MPTP 3w=20 | 7.60±0.20 |  |  | WT+Saline vs *Drd2*-Cdc42KO+Saline, p<0.01 *** |
|  |  | *Drd2*-Cdc42KO+MPTP 3w=21 | 7.39±0.37 |  |  |  |
| Figure 6h | Thin spine density | WT+Saline=25 | 7.63±0.14 | Two-way ANOVA | Genotype, F (1, 144) =58.616, p<0.001 ***  Drug treatment, F (2, 144) =83.722, p<0.001 ***  Genotype x Drug treatment, F (2, 144) =38.736, p<0.001 *** | WT+Saline vs WT+MPTP 48h, p<0.01 *** |
|  |  | *Drd2*-Cdc42KO+Saline=27 | 4.46±0.13 |  |  | WT+Saline vs WT+MPTP 3w, p<0.01 *** |
|  |  | WT+MPTP=27 | 3.96±0.14 |  |  | *Drd2*-Cdc42KO+Saline vs *Drd2*-Cdc42KO+MPTP 48h, p<0.05 * |
|  |  | *Drd2*-Cdc42KO+MPTP=24 | 3.71±0.31 |  |  | WT+Saline vs *Drd2*-Cdc42KO+Saline, p<0.01 *** |
|  |  | WT+MPTP 3w=20 | 4.11±0.15 |  |  |  |
|  |  | *Drd2*-Cdc42KO+MPTP 3w=21 | 3.85±0.24 |  |  |  |
| Figure 6i | Mushroom spine density | WT+Saline=25 | 3.76±0.17 | Two-way ANOVA | Genotype, F (1, 144) =1.926, p=0.168  Drug treatment, F (2, 144) =51.369, p<0.001 ***  Genotype x Drug treatment, F (2, 144) =0.456, p=0.501 |  |
|  |  | *Drd2*-Cdc42KO+Saline=27 | 3.48±0.14 |  |  |  |
|  |  | WT+MPTP=27 | 2.68±0.14 |  |  |  |
|  |  | *Drd2*-Cdc42KO+MPTP=24 | 2.58±0.09 |  |  |  |
|  |  | WT+MPTP 3w=20 | 2.55±0.15 |  |  |  |
|  |  | *Drd2*-Cdc42KO+MPTP 3w=21 | 2.67±0.15 |  |  |  |
| Figure 6j | Stubby spine density | WT+Saline=25 | 0.93±0.06 | Two-way ANOVA | Genotype, F (1, 144) =1.357, p=0.246  Drug treatment, F (2, 144) =0.693, p=0.502  Genotype x Drug treatment, F (2, 144) =0.342, p=0.711 |  |
|  |  | *Drd2*-Cdc42KO+Saline=27 | 0.79±0.07 |  |  |  |
|  |  | WT+MPTP=27 | 0.87±0.07 |  |  |  |
|  |  | *Drd2*-Cdc42KO+MPTP=24 | 0.83±0.05 |  |  |  |
|  |  | WT+MPTP 3w=20 | 0.95±0.09 |  |  |  |
|  |  | *Drd2*-Cdc42KO+MPTP 3w=21 | 0.92±0.08 |  |  |  |
| **Figure** | **Measurement** | **Number of mice** | **Values** | **Statistical Test** | **Treatments (p value)** | **simple main effect (p value)** |
| Figure 6k | Overall Rotarod Performance | WT+Saline=8 | 1978.75±47.76 | Two-way ANOVA | Genotype, F (1, 26) =1.331, p=0.261  Drug treatment, F (1, 26) =21.159, p<0.001 ***  Genotype x Drug treatment, F (1, 26) =1.183, p=0.289 |  |
|  |  | *Drd2*-Cdc42KO+Saline=6 | 1735.83±95.46 |  |  |  |
|  |  | WT+MPTP=6 | 1362.33±157.04 |  |  |  |
|  |  | *Drd2*-Cdc42KO+MPTP=6 | 1355.17±130.75 |  |  |  |
| Figure 6l | Latency to turn around in the pole test | WT+Saline=8 | 3.38±0.18 | Two-way ANOVA | Genotype, F (1, 28) =8.201, p=0.009 **  Drug treatment, F (1, 28) =27.440, p<0.001 ***  Genotype x Drug treatment, F (1, 28) =1.942, p=0.176 | WT+Saline vs WT+MPTP, p<0.001 *** |
|  |  | *Drd2*-Cdc42KO+Saline=8 | 4.63±0.37 |  |  | WT+Saline vs *Drd2*-Cdc42KO+Saline, p<0.001 *** |
|  |  | WT+MPTP=6 | 5.33±0.23 |  |  |  |
|  |  | *Drd2*-Cdc42KO+MPTP=6 | 5.76±0.42 |  |  |  |
| Figure 6m | Latency to land on the ground in the pole test | WT+Saline=8 | 5.97±0.32 | Two-way ANOVA | Genotype, F (1, 28) =22.514, p<0.001 ***  Drug treatment, F (1, 28) =43.470, p<0.001 ***  Genotype x Drug treatment, F (1, 28) =18.811, p<0.001 *** | WT+Saline vs WT+MPTP, p<0.001 *** |
|  |  | *Drd2*-Cdc42KO+Saline=8 | 10.67±0.71 |  |  | WT+Saline vs *Drd2*-Cdc42KO+Saline, p<0.001 *** |
|  |  | WT+MPTP=6 | 11.62±0.42 |  |  |  |
|  |  | *Drd2*-Cdc42KO+MPTP=6 | 11.83±0.65 |  |  |  |
| Figure 6n | The percentage of time exploring the novel object in the NOR task | WT+Saline=9 | 82.22±1.98 | Two-way ANOVA | Genotype, F (1, 27) =51.036, p<0.001 ***  Drug treatment, F (1, 27) =71.629, p<0.001 ***  Genotype x Drug treatment, F (1, 27) =41.683, p<0.001 *** | WT+Saline vs WT+MPTP, p<0.001 *** |
|  |  | *Drd2*-Cdc42KO+Saline=6 | 56.88±1.60 |  |  | WT+Saline vs *Drd2*-Cdc42KO+Saline, p<0.001 *** |
|  |  | WT+MPTP=6 | 54.42±1.57 |  |  |  |
|  |  | *Drd2*-Cdc42KO+MPTP=6 | 53.14±1.73 |  |  |  |

**Table S13. Statistical analysis in Figure S1.**

| **Figure** | **Measurement** | **Number of mice** | **Values** | **Statistical Test** | **Treatments (p value)** |
| --- | --- | --- | --- | --- | --- |
| Figure S1b | Active Cdc42 | WT =3 | 1.00±0.03 | One-way ANOVA | F (3, 11) =0.606, p=0.630 |
|  |  | MPTP 0.5 h=3 | 1.09±0.12 |  |  |
|  |  | MPTP 2 h=3 | 0.93±0.06 |  |  |
|  |  | MPTP 24 h=3 | 0.94±0.14 |  |  |
| Figure S1c | Total Cdc42 | WT =3 | 1.00±0.50 |  | F (3, 11) =0.485, p=0.702 |
|  |  | MPTP 0.5 h=3 | 0.96±0.15 |  |  |
|  |  | MPTP 2 h=3 | 0.85±0.18 |  |  |
|  |  | MPTP 24 h=3 | 1.09±0.16 |  |  |

**Table S14. Statistical analysis in Figure S2.**

| **Figure** | **Measurement** | **Number of mice** | **Values** | **Statistical Test** | **Treatments (p value)** | **simple main effect (p value)** |
| --- | --- | --- | --- | --- | --- | --- |
| Figure S2c | Active Cdc42 | LV-EGFP=4 | 1.00±0.10 | One-way ANOVA | F (2, 11) =58.845, p<0.001 *** | LV-EGFP vs Cdc42-ca, p<0.001 ***;  LV-EGFP vs Cdc42-dn, p=0.032 * |
|  |  | Cdc42-ca=4 | 2.29±0.10 |  |  |  |
|  |  | Cdc42-dn=4 | 0.44±0.16 |  |  |  |
| Figure S2d | Total Cdc42 | LV-EGFP=4 | 1.00±0.03 |  | F (2, 11) =0.751, p=0.499 |  |
|  |  | Cdc42-ca=4 | 0.97±0.13 |  |  |  |
|  |  | Cdc42-dn=4 | 0.84±0.10 |  |  |  |
| Figure S2e | Phosphorylation of NWASP | LV-EGFP=4 | 1.00±0.15 |  | F (2, 11) =40.113, p<0.001 *** | LV-EGFP vs Cdc42-ca, p<0.001 ***;  LV-EGFP vs Cdc42-dn, p=0.048 * |
|  |  | Cdc42-ca=4 | 2.32±0.23 |  |  |  |
|  |  | Cdc42-dn=4 | 0.34±0.03 |  |  |  |
| Figure S2f | NWASP | LV-EGFP=3 | 1.00±0.12 |  | F (2, 8) =0.006, p=0.994 |  |
|  |  | Cdc42-ca=3 | 0.98±0.19 |  |  |  |
|  |  | Cdc42-dn=3 | 0.97±0.19 |  |  |  |
| Figure S2g | Phosphorylation of PAK | LV-EGFP=4 | 1.00±0.08 |  | F (2, 11) =32.113, p<0.001 *** | LV-EGFP vs Cdc42-ca, p=0.002 **;  LV-EGFP vs Cdc42-dn, p=0.047 * |
|  |  | Cdc42-ca=4 | 2.04±0.24 |  |  |  |
|  |  | Cdc42-dn=4 | 0.38±0.06 |  |  |  |
| Figure S2h | PAK | LV-EGFP=4 | 1.00±0.08 |  | F (2, 11) =0.229, p=0.800 |  |
|  |  | Cdc42-ca=4 | 1.02±0.10 |  |  |  |
|  |  | Cdc42-dn=4 | 1.09±0.11 |  |  |  |

**Table S15. Statistical analysis in Figure S4.**

| **Figure** | **Measurement** | **Number of mice** | **Values** | **Statistical Test** | **Treatments (p value)** |
| --- | --- | --- | --- | --- | --- |
| Figure S4b | D2R | WT=4 | 1.00±0.13 | Unpaired two-tailed t-test | t =5.030, p=0.002 ** |
|  |  | CPu-*Drd2*KO=4 | 0.30±0.05 |  |  |
| Figure S4d | Cdc42 | WT=4 | 1.00±0.09 | Unpaired two-tailed t-test | t =4.682, p=0.003 ** |
|  |  | *Drd2*-Cdc42KO=4 | 0.47±0.07 |  |  |

**Table S16. Statistical analysis in Figure S5.**

| **Figure** | **Measurement** | **Number of mice** | **Values** | **Statistical Test** | **Treatments (p value)** | **simple main effect (p value)** |
| --- | --- | --- | --- | --- | --- | --- |
| Figure S5c | Overall Rotarod Performance | WT =7 | 1994.71± 81.35 | One-way ANOVA | F (2, 20) =41.344, p<0.001 *** | WT vs MPTP, p<0.001 ***;  MPTP vs MPTP+quinpirole, p<0.001 ### |
|  |  | MPTP=7 | 1038.71±70.42 |  |  |  |
|  |  | MPTP+quinpirole=7 | 1789.71±82.50 |  |  |  |
| Figure S5d | Latency to turn around in the pole test | WT =7 | 1.41±0.12 |  | F (2, 20) =17.306, p<0.001 *** | WT vs MPTP, p<0.001 ***;  MPTP vs MPTP+quinpirole, p<0.001 ### |
|  |  | MPTP=7 | 3.19±0.39 |  |  |  |
|  |  | MPTP+quinpirole=7 | 1.40±0.14 |  |  |  |
| Figure S5e | Latency to land on the ground in the pole test | WT =7 | 6.80±0.62 |  | F (2, 20) =14.645, p<0.001 *** | WT vs MPTP, p=0.001 **;  MPTP vs MPTP+quinpirole, p<0.001 ### |
|  |  | MPTP=7 | 11.73±1.13 |  |  |  |
|  |  | MPTP+quinpirole=7 | 6.07±0.54 |  |  |  |
| Figure S5f | The percentage of time exploring the novel object in the NOR task | WT =6 | 74.40±4.35 |  | F (2, 18) =14.374, p<0.001 *** | WT vs MPTP, p<0.001 ***;  MPTP vs MPTP+quinpirole, p=0.027 ## |
|  |  | MPTP=7 | 41.11±5.10 |  |  |  |
|  |  | MPTP+quinpirole=6 | 59.62±3.44 |  |  |  |

**Table S17. Statistical analysis in Figure S6.**

| **Figure** | **Measurement** | **Number of spines** | **Values** | **Statistical Test** | **Treatments (p value)** |
| --- | --- | --- | --- | --- | --- |
| Figure S6d | Total spine density | D1-MSN WT+Saline=20 | 12.86±0.55 | Two-way ANOVA | Genotype, F (1, 77) =0.013 p=0.910  Drug treatment, F (1, 77) =74.512, p<0.001 ***  Genotype x Drug treatment, F (1, 77) =0.002, p=0.926 |
|  |  | D1-MSN CPu-*Drd2*KO+Saline=18 | 12.80±0.29 |  |  |
|  |  | D1-MSN WT+MPTP=20 | 9.56±0.29 |  |  |
|  |  | D1-MSN CPu-*Drd2*KO+MPTP=19 | 9.54±0.28 |  |  |
| Figure S6e | Thin spine density | D1-MSN WT+Saline=20 | 7.89±0.27 | Two-way ANOVA | Genotype, F (1, 77) =0.722 p=0.398  Drug treatment, F (1, 77) =69.712, p<0.001 ***  Genotype x Drug treatment, F (1, 77) =2.710, p=0.104 |
|  |  | D1-MSN CPu-*Drd2*KO+Saline=18 | 8.10±0.29 |  |  |
|  |  | D1-MSN WT+MPTP=20 | 6.13±0.23 |  |  |
|  |  | D1-MSN CPu-*Drd2*KO+MPTP=19 | 5.47±0.26 |  |  |
| Figure S6f | Mushroom spine density | D1-MSN WT+Saline=20 | 3.83±0.24 | Two-way ANOVA | Genotype, F (1, 77) =1.372 p=0.245  Drug treatment, F (1, 77) =20.008, p<0.001 ***  Genotype x Drug treatment, F (1, 77) =2.814, p=0.098 |
|  |  | D1-MSN CPu-*Drd2*KO+Saline=18 | 3.72±0.21 |  |  |
|  |  | D1-MSN WT+MPTP=20 | 2.50±0.20 |  |  |
|  |  | D1-MSN CPu-*Drd2*KO+MPTP=19 | 3.12±0.21 |  |  |
| Figure S6g | Stubby spine density | D1-MSN WT+Saline=20 | 1.14±0.17 | Two-way ANOVA | Genotype, F (1, 77) =0.467 p=0.496  Drug treatment, F (1, 77) =1.111, p=0.295  Genotype x Drug treatment, F (1, 77) =0.680, p=0.412 |
|  |  | D1-MSN CPu-*Drd2*KO+Saline=18 | 0.97±0.10 |  |  |
|  |  | D1-MSN WT+MPTP=20 | 0.93±0.05 |  |  |
|  |  | D1-MSN CPu-*Drd2*KO+MPTP=19 | 0.95±0.05 |  |  |
